# Exposure to dim light at night alters daily rhythms of glucose and lipid metabolism in rats

**DOI:** 10.3389/fphys.2022.973461

**Published:** 2022-08-29

**Authors:** Valentina Sophia Rumanova, Monika Okuliarova, Ewout Foppen, Andries Kalsbeek, Michal Zeman

**Affiliations:** ^1^ Department of Animal Physiology and Ethology, Faculty of Natural Sciences, Comenius University, Bratislava, Slovakia; ^2^ Hypothalamic Integration Mechanisms, Netherlands Institute for Neuroscience (NIN), An Institute of the Royal Netherlands Academy of Arts and Sciences (KNAW), Amsterdam, Netherlands; ^3^ Laboratory of Endocrinology, Amsterdam UMC, Amsterdam Gastroenterology Endocrinology Metabolism (AGEM), Amsterdam, Netherlands; ^4^ Department of Endocrinology and Metabolism, Amsterdam UMC, University of Amsterdam, Amsterdam, Netherlands

**Keywords:** dim light at night, daily rhythms, metabolism, metabolic sensors, peripheral clocks, locomotor activity, substrate oxidation

## Abstract

Nocturnal light pollution has been rapidly increasing during the last decades and even though dim artificial light at night (ALAN) has been associated with metabolic diseases, its mechanism is still far from clear. Therefore, the aim of our study was to thoroughly analyze the effects of ALAN on energy metabolism, metabolites, metabolic hormones, and gene expression. Male Wistar rats were kept in either the standard light:dark (12:12) cycle or exposed to ALAN (∼2 lx) during the whole 12-h dark phase for 2 weeks. Energy metabolism was measured in metabolic cages. In addition, we measured plasma and hepatic metabolites, clock and metabolic gene expression in the liver and epididymal adipose tissue, and plasma hormone levels. In ALAN rats, we observed an unexpected transitory daytime peak of locomotor activity and a suppression of the peak in locomotor activity at the beginning of the dark period. These changes were mirrored in the respiratory exchange ratio. Plasma metabolites became arrhythmic, and plasma and hepatic cholesterol levels were increased. Lost rhythmicity of metabolites was associated with disrupted behavioral rhythms and expression of metabolic genes. In the liver, the rhythms of metabolic sensors were either phase-advanced (*Ppara, Pgc1a, Nampt*) or arrhythmic (*Sirt1, Lxra*) after ALAN. The rhythmic pattern of *Ppara* and *Sirt1* was abolished in the adipose tissue. In the liver, the amplitude of the daily rhythm in glycogen content was attenuated, the *Glut2* rhythm was phase-advanced and *Foxo1* lost its daily rhythmicity. Moreover, hepatic *Foxo1* and *Gck* were up-regulated after ALAN. Interestingly, several parameters of lipid metabolism gained rhythmicity (adiponectin, *Hmgcs2, Lpl, Srebf1c*) in the liver, whereas *Noct* became arrhythmic in the adipose tissue. Peripheral clock genes maintained their robust oscillations with small shifts in their acrophases. Our data show that even a low level of ALAN can induce changes in the daily pattern of behavior and energy metabolism, and disturb daily rhythms of genes encoding key metabolic sensors and components of metabolic pathways in the liver and adipose tissue. Disturbed metabolic rhythms by ALAN could represent a serious risk factor for the development and progression of metabolic diseases.

## Introduction

The ability of an organism to anticipate the daily changes in the environment and synchronize responses of different organs and tissues of the body with these changes is mediated by the internal circadian clock. The circadian system is hierarchically organized with the central oscillator located in the suprachiasmatic nuclei (SCN) of the hypothalamus synchronizing the peripheral clocks present in every cell of the body (Bechtold and Loudon, 2013). The SCN regulates circadian rhythms in the periphery directly via humoral and neuronal pathways but also indirectly, through behavioral signals, ensuring that all organs in the body act in synchrony with the environmental conditions (Kalsbeek et al., 2006).

The generation of circadian rhythms is based on the transcriptional-translational feedback loop, which consists of the positive (CLOCK—circadian locomotor output cycles protein kaput, and BMAL1—brain and muscle ARNT-like 1) and negative elements (PER—periods, and CRY—cryptochromes). In the nucleus, the heterodimer CLOCK:BMAL1 promotes the transcription of *Per* and *Cry* and this negative limb of the loop, in return, suppresses its own transcription by the interaction of PER:CRY with CLOCK:BMAL1. The process is endogenous and lasts approximately 24 h (Cox and Takahashi, 2019). Moreover, the main loop is strengthened by several accessory feedback loops. One of the most important accessory loops includes the nuclear receptor subfamily 1, group D, member 1 (*Nr1d1* or REV-ERBα), which is a negative regulator of *Bmal1* transcription and competes with the RAR-related orphan receptor (ROR) at the ROR-response element of the *Bmal1* gene promoter (Preitner et al., 2002). In addition, REV-ERBα plays a major role in the circadian control of genes involved in the metabolism (Cho et al., 2012; Ikeda et al., 2019). The molecular clock and metabolism have a mutual bidirectional relationship since the clock regulates the transcription of metabolic genes and in return, metabolites can affect the molecular clockwork (Reinke and Asher, 2019).

In addition to clock and clock-controlled genes, rhythms of metabolic sensors are important for the stability and fine-tuning of the clockwork to the metabolic status (Dyar et al., 2018; Reinke and Asher, 2019). Circadian clocks help to prepare an organism for daily changes in food intake, ensuring that proper metabolic processes are ready to respond to different metabolic needs during fasting or feeding. The ability of the clock to adapt to alterations in metabolic state (e.g. due to altered feeding pattern) is enabled by metabolic sensors that provide feedback needed for the necessary changes. During energy depletion, adenosine monophosphate (AMP) accumulates inside the cell and activates AMP-activated protein kinase (AMPK). This enzyme promotes CRY degradation and controls the transcription of nicotinamide phosphoribosyltransferase (*Nampt*) through its effects on the clock (Lee and Kim, 2013). *Nampt* is a key player in the salvation of nicotinamide adenine dinucleotide (NAD^+^) (Reinke and Asher, 2019; Van Der Spek et al., 2021), the cofactor of sirtuin 1 (SIRT1). SIRT1, with the help of NAD^+^, deacetylates enzymes of catabolic processes but also affects the main loop of the molecular clock (Nakahata et al., 2008; Levine et al., 2020).

For optimal performance and health, a proper alignment of bodily circadian rhythms with behavior and the environmental light:dark (LD) cycle is necessary. The central circadian oscillator in the hypothalamus is entrained predominantly by the LD cycle, but behavioral rhythms in food intake and sleep/activity are important for the synchronization of the peripheral clocks (Chaix et al., 2019). Misalignment of the circadian rhythms among different organs or their desynchronization from the environment increases the risk for cardiovascular disease, diabetes, obesity, cancer, and psychiatric disorders (Bae et al., 2019). Such disruptions of the precise time organization of the body can for instance be caused by shift work (Boivin et al., 2022), jet lag (Paragliola et al., 2021), night-time eating (Riccobono et al., 2020), or exposure to artificial light at night (ALAN) (Bumgarner and Nelson, 2021). Nocturnal light pollution has been increasing at an alarming rate during the last 2 decades (Cinzano et al., 2001; Falchi et al., 2016) and can have detrimental consequences on ecosystems (Grubisic et al., 2019) and health (Stevens and Zhu, 2015).

Studies in both rodents and humans indicate that ALAN can interfere with daily rhythms of activity and metabolism. In Swiss Webster mice, exposure to 5 lux during the dark-time resulted in the development of obesity accompanied by elevated daytime food intake and the impairment of glucose tolerance (Fonken et al., 2010; Fonken et al., 2013). In rats, similar lighting conditions did not affect body weight, but did disturb the circadian control of sleep (Stenvers et al., 2016) and deposition of lipids to the liver (Rumanova et al., 2019; Okuliarova et al., 2020). In our recent study, we showed that dim ALAN suppresses daily rhythms of clock genes and vasopressin in the central oscillator and resulted in altered rhythms in hormonal outputs, and food and water intake (Okuliarova et al., 2022). Since we assumed that disruption of circadian rhythms of physiological and behavioral processes plays a key role in mediating the negative metabolic effects of light pollution, here, we explored how ALAN affects the temporal coordination of metabolic pathways in the liver and adipose tissue.

The aim of our study was to investigate the daily pattern of locomotor and metabolic activity in rats kept in metabolic cages either under the standard 12:12 LD cycle or after 2-week exposure to low-intensity artificial light (ALAN) during the whole 12-h dark phase, i.e., conditions of the attenuated central oscillator induced by all-night exposure to low-intensity light, such as usually present in urban areas.

In addition, we explored rhythmic changes of key metabolites and metabolic hormones during the 24-h period. To better understand molecular mechanisms determining metabolic responses to ALAN exposure, we analyzed alterations in daily oscillations of clock genes, metabolic sensors, and genes encoding enzymes and transcription factors involved in the regulation of glucose and lipid metabolism.

## Materials and methods

### Ethics approval statement

The first experiment was in agreement with Dutch laws and the Council Directive 2010/63EU of the European Parliament and the Council of 22 September 2010 on the protection of animals used for scientific purposes. All procedures were also approved by the Animal Ethics Committee of the Royal Dutch Academy of Arts and Sciences (KNAW, Amsterdam, Netherlands) and in accordance with the guidelines on animal experimentation of the Netherlands Institute for Neuroscience. The second experiment was approved by the Ethical Committee for the Care and Use of Laboratory Animals at the Comenius University in Bratislava, Slovak Republic and the State Veterinary Authority of the Slovak Republic (Ro-1648/19-221/3).

### Experiment 1

#### Metabolic cages

In the first experiment, adult male Wistar WU rats (*n* = 16; Charles River, Sulzfeld, Germany) were individually housed in Metabolic PhenoCages (TSE Systems, Bad Hombourg, Germany) for the entire duration of the experiment. Animals were kept in clear plastic cages and were acclimatized to the change in environment under the standard 12:12 LD cycle with Zeitgeber Time 0 (ZT0, lights on) at 7:00 in the morning. Cages were placed in the climate-controlled chamber with stable ambient temperature of 23.5 ± 0.2 and humidity of 55–65%. Water and standard chow (2918, Envigo, Horst, Netherlands) were available *ad libitum*. After 1 week of accommodation, rats (314 ± 5 g) were assigned to the control group (CTRL, *n* = 8), which remained in the same standard lighting regime, or the experimental group (ALAN, *n* = 8) exposed to the dim light throughout the whole 12-h night. The light was emitted by LED strips mounted around the cages. During the light phase, light reached the illumination of ∼150 lx and color temperature between 2,700-3,000 K with a peak wavelength of 610 nm. More information about the spectral power distribution of the different light sources used can be found in Supplementary Figures S1C,D. Throughout the dark phase, the ALAN group was exposed to light within the range of 2–3.5 lx. Locomotor activity, O_2_ consumption (VO_2_), and CO_2_ production (VCO_2_) per rat were measured by indirect calorimetry (PhenoMaster, TSE Systems, Bad Hombourg, Germany). Data were collected in 15-min intervals throughout the whole experiment, except for the time when replacing food and water twice a week, and the weekly cleaning of cages when the measurements were paused. For the analysis, we used three consecutive undisturbed days of the second experimental week. The respiratory exchange ratio (RER) was calculated by the software according to the commonly used formula VCO_2_/VO_2_.

#### EchoMRI

To analyze body composition, animals were weighed and scanned using an EchoMRI (EchoMRI™, Houston, TX, United States) once a week during the cleaning of metabolic cages. Fat and lean mass was normalized to body weight.

### Experiment 2

#### Animals and experimental design

Adult male Wistar rats (*n* = 72) were obtained from the breeding station at the Institute of Experimental Pharmacology and Toxicology, Slovak Academy of Sciences (Dobrá Voda, Slovak republic). Animals were housed in transparent plastic cages (four rats per cage) under controlled stable conditions (ambient temperature of 21.5 ± 1.3°C and humidity of 55–65%) with *ad libitum* access to standard chow (MP-OŠ-06, Peter Miško, Snina, Slovakia) and drinking water. Animals were adapted to the 12:12 LD cycle with ZT0 at 6:00 in the morning for 2 weeks. A warm light bulb (2,900 K) was used as the source of light. During the daytime, light illumination was in the range of 150—200 lx. The characteristics of the spectral power distribution of the light sources used are shown in Supplementary Figure S2. After 2 weeks of acclimatization, animals (275 ± 3 g) were assigned to the CTRL or ALAN group as described above. The CTRL group (*n* = 36) remained in the original LD regime and the ALAN group (*n* = 36) was exposed to low-intensity light with the illumination of ∼2 lx during the night-time.

#### Sample collection

Rats were sacrificed by decapitation under isoflurane anesthesia (induction time: 15–45 s) every 4 hours (ZT6, ZT10, ZT14, ZT18, ZT22, ZT2). Trunk blood was collected into the tubes containing either heparin or EDTA with aprotinin. Plasma was separated after centrifugation (2,500xg, 10 min, 4°C). The liver and epididymal adipose tissue were snap-frozen in liquid nitrogen and stored at −80°C until further analysis.

#### Plasma metabolites

Plasma concentrations of glucose, triacyglycerols, cholesterol and LDL-cholesterol were measured enzymatically using commercial kits BIO-LA-TEST (Erba Lachema, Brno, Czech Republic) according to the manufacturer’s instructions.

#### Hepatic lipid and glycogen extraction

Hepatic lipids were extracted by the Folch method (Folch et al., 1957). Briefly, the liver was homogenized in the mixture of chloroform:methanol (2:1). After incubation and centrifugation, we separated polar and nonpolar compounds by adding 0.9% NaCl. The bottom chloroform phase containing triacylglycerols and cholesteryl esters was transferred into clean tubes and evaporated under the stream of nitrogen. Samples were resuspended in isopropanol and isolated lipids (triacylglycerols, cholesterol) were measured in the same way as described above.

Glycogen extraction was performed as previously described (Sullivan et al., 2014). The liver was homogenized in Tris-buffered saline (TBS) with phosphatase inhibitors. Samples were incubated in ammonium acetate buffer, one series contained amyloglucosidase and the second (control) series did not. Amyloglucosidase breaks down glycogen to glucose units that were quantified by commercial kits BIO-LA-TEST (Erba Lachema, Brno, Czech Republic). The purpose of the control series was to determine the amount of glucose that is not stored in the form of glycogen. The glucose values from the control series were subtracted from the values of samples containing amyloglucosidase. The results were used to calculate the amount of glycogen (mg) per gram of tissue.

#### Hormone assays

Plasma levels of insulin, leptin and adiponectin were measured using the following enzyme-linked immunosorbent assay (ELISA) kits: Rat Insulin ELISA (EZRMI-13K; Merck Millipore, Burlington, MA, United States), Rat Leptin ELISA (RD291001200R; BioVendor, Brno, Czech Republic) and Rat Adiponectin ELISA (EZRADP-62K; Merck Millipore, Burlington, MA, United States), according to the manufacturer’s instructions. Intra-assay variation coefficients were lower than 5% for all assays.

#### RT-qPCR

The liver and adipose tissue were homogenized using FastPrep (M.P. Biochemicals, Irvine, CA, United States) and tubes with 1.4 mm ceramic spheres (M.P. Biochemicals, Irvine, CA, United States). Total RNA was isolated by guanidine-tiocyanat-phenol-chloroform extraction with TRI reagent (Molecular Research Center, Cincinnati, OH, United States). Extracted hepatic RNA was purified using ice-cold isopropanol and 5 M ammonium acetate. We used RNeasy Plus universal Mini kit (Qiagen, Düsseldorf, Germany) to isolate RNA from epididymal adipose tissue. The concentration and purity of RNA were measured by NanoDrop One (Thermo Fisher Scientific, Waltham, MA, United States), while the integrity was verified by electrophoresis using 1.2% agarose gel. Any genomic DNA present in samples was eliminated using DNase I (Thermo Fisher Scientific, Waltham, MA, United States). Complementary DNA was transcribed from 1,000 ng/μL of isolated RNA using Maxima cDNA synthesis kit (Thermo Fisher Scientific, Waltham, MA, United States). The concentration of transcribed cDNA was 10 ng/μL. The final concentration of diluted DNA used for qPCR was 1 ng/μL. Real-time PCR was performed using Maxima SYBR Green qPCR Master Mix (Thermo Fisher Scientific, Waltham, MA, United States) and CFX Connect real-time PCR detection system (Bio-Rad Laboratories, Hercules, CA, United States). The gene expression was calculated using a standard curve method. Expression levels of target genes were normalized to the levels of reference genes, β-2-microglobulin (*B2m*) and 18S ribosomal RNA (*Rn18s*) in the liver and adipose tissue, respectively. All used primers are listed in the Supplementary Table S1.

#### Western blot method

The samples of liver were homogenized on ice by ULTRA-TURRAX (IKA, Staufen, Germany) in 0.3 M sucrose buffer (pH = 7) in the presence of protease and phosphatase inhibitors (sodium fluoride, sodium orthovanadate). Protein concentration was measured by the BCA assay (Merck Millipore, Burlington, MA, United States) according to the manufacturer’s instructions. Total proteins (40 or 80 µg) were separated by SDS-PAGE electrophoresis at 60 V for 20 min and then at 90 V for 80 min. Proteins were transferred to a nitrocellulose membrane using the vertical apparatus (Bio-Rad Laboratories, Hercules, CA, United States) at 230 mA for 75 min. Membranes were blocked by 1% fat-free milk or 5% bovine serum albumin (BSA) in TBS containing Tween 20 (TBS-T) for 1 h at room temperature. Subsequently, membranes were incubated with a primary antibody overnight at 4°C: anti-AMP-activated protein kinase α (2603, AMPKα; Cell Signaling Technology, Danvers, MA, United States, 1:1,000 dilution in 1% fat-free milk in TBS-T), anti-phospho-AMPKα (4188, Cell Signaling Technology, Danvers, MA, United States, 1:1,000 dilution in 1% fat-free milk in TBS-T), anti-sirtuin 1 (9475, Cell Signaling Technology, Danvers, MA, United States, 1:500 dilution in 1% BSA in TBS-T) and anti-REV-ERBα (ab174309, Abcam, Cambridge, United Kingdom, 1:1,000 dilution in 1% BSA in TBS-T). After thorough washing (3 min × 10 min) in TBS-T, membranes were incubated with anti-rabbit horseradish peroxidase-conjugated secondary antibody (7074, Cell Signaling Technology, Danvers, MA, United States, 1:2,000 dilution in 1% fat-free milk in TBS-T) for 1 h at RT. All membranes were stripped, blocked with 5% fat-free milk in TBS-T, and incubated with anti-glyceraldehyde-3-phosphate (MAB374, GAPDH, clone 6C5, Merck Millipore, Burlington, MA, United States, 1:5,000 dilution in 1% fat-free milk in TBS-T) for 1 h at RT. Subsequently, membranes were washed and incubated with anti-mouse horseradish peroxidase-conjugated secondary antibody (7076, Cell Signaling Technology, Danvers, MA, United States, 1:2,000 dilution in 1% fat-free milk in TBS-T) for 1 h at RT. Proteins were visualized using Clarity Western ECL substrate (Bio-Rad Laboratories, Hercules, CA, United States) and chemiluminescence imaging system Vü-C (Pop-Bio Imaging, Cambridge, United Kingdom). Protein expression was quantified by software Image Studio Lite (LI-COR Biosciences, Lincoln, NE, United States) and signals from target proteins were normalized to GAPDH.

#### Statistical analysis

Data were analyzed using R software (R Core Team, 2020). A cosinor analysis was performed using packages “cosinor” and “cosinor2” that fit a cosine curve to the data by a linear least-squares regression method. The significant 24-h rhythm was detected with an F-test by rejection of the zero-amplitude hypothesis. In a case of a significant rhythm, cosinor analysis provided us with the mesor (the 24-h time series mean), amplitude (one-half the peak-to-trough difference) and acrophase (the peak time of the fitted curve). Differences between acrophases and amplitudes of rhythmic parameters between CTRL and ALAN groups were analyzed by Wald tests. To reveal significant effect of ALAN, we further examined data of daily rhythms and protein expression using two-way analysis of variance (ANOVA, factors: ZT and ALAN). Body composition data, locomotor activity and RER in 1-h intervals and mean 12-h values were analyzed using two-way repeated measures ANOVA (factors: Week and ALAN, LD and ALAN or ZT and ALAN). In the case of significant interaction of factors, we used Fisher’s LSD or Bonferroni’s multiple comparisons *post hoc* test. Differences between groups in mean 24-h values of the locomotor activity and RER were analyzed by the Student’s t-test. A *p*-value of <0.05 was considered to be statistically significant. All data are expressed as the mean ± standard error of mean (SEM). Results from cosinor analysis and two-way ANOVA are available in Supplementary Tables S2–S10.

## Results

### ALAN increases daytime locomotor activity and alters substrate oxidation

**FIGURE 1:**
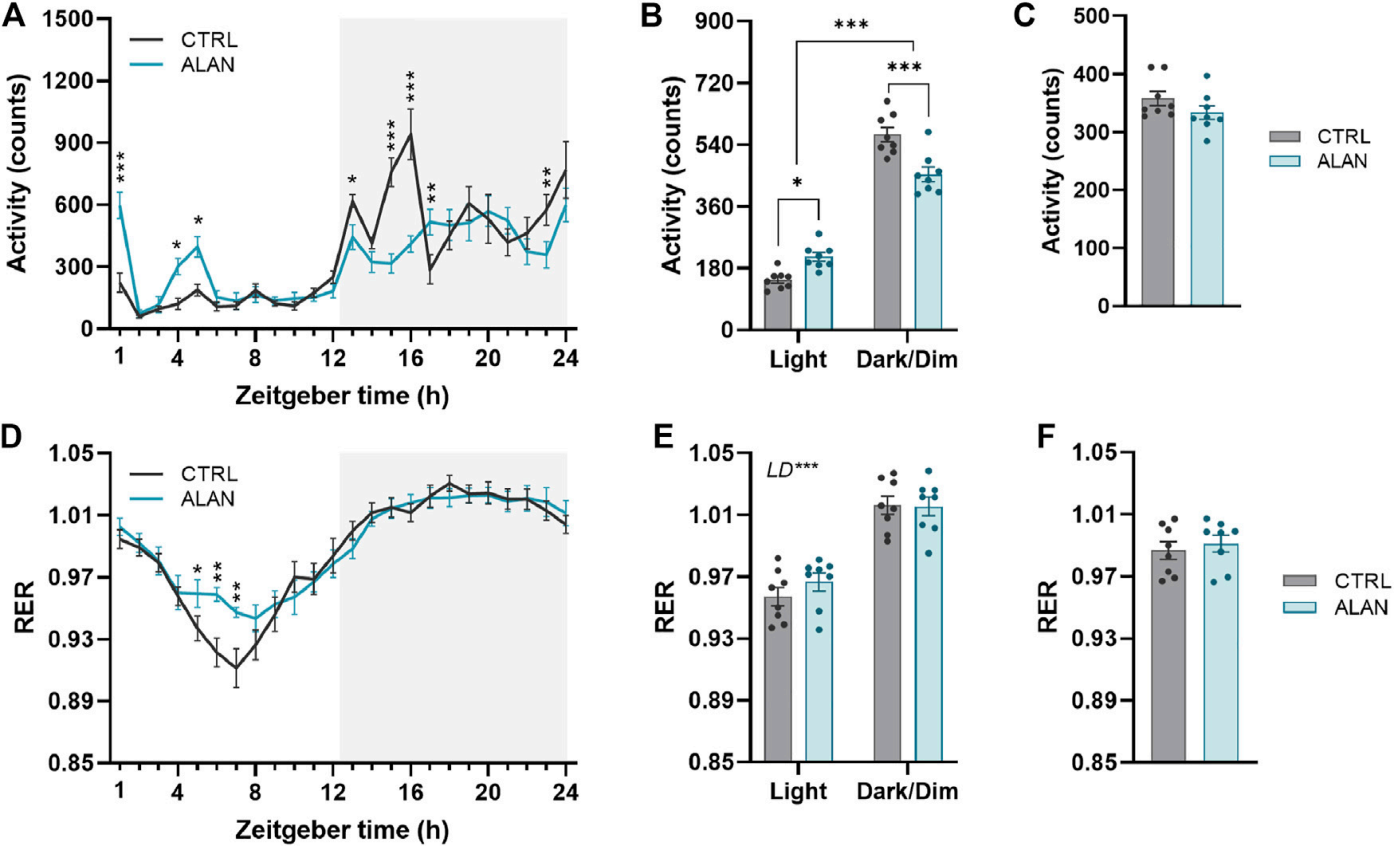
Dim artificial light at night (ALAN) changes the daily rhythm of locomotor activity **(A–C)** and respiratory exchange ratio (RER, **(D–F)**. Rats were kept either in the standard LD regime (CTRL; grey) or exposed to dim artificial light at night (ALAN; blue) for 2 weeks. Daily rhythms of parameters are presented in 1-h intervals over 24 h **(A,D)**. Mean values of parameters per 12 h (light and dark/dim light phase) **(B,E)**. Mean 24-h values of measured parameters **(C,F)**. The grey area indicates the dark/dim light phase. Data represent mean ± SEM with *n* = 8 per group. Two-way ANOVA with repeated measures with subsequent Fisher’s LSD or Bonferroni’s *post hoc* test was used to compare groups (CTRL and ALAN) in 1-h intervals or during the light and dark/dim light phase, respectively. 24-h mean data were analyzed by the Student’s t-test to reveal differences between groups. Significant effects are indicated by *p < 0.05, **p < 0.01, ***p < 0.001).

The amplitude of the locomotor activity rhythm in the CTRL group was dampened by ALAN exposure (*p* < 0.01; Figure 1A; Supplementary Table S2). The characteristic bimodal pattern of locomotor activity with rises at the beginning and end of the active period was changed by ALAN exposure. The two-way ANOVA and subsequent post hoc test revealed that the peak at the beginning of the night (ZT13—ZT17) found in controls, was suppressed after ALAN exposure (ZT13: *p* < 0.05, ZT15: *p* < 0.001, ZT16: *p* < 0.001, ZT17: *p* < 0.01; Supplementary Table S2). On the other hand, a smaller extra peak of locomotor activity was observed during the light period in the ALAN group (ZT1: *p* < 0.001, ZT4: *p* < 0.05, ZT5: *p* < 0.05; Supplementary Table S2). Actograms are shown in Supplementary Figures S3, S4. This change in the daily pattern (*F*_(1,14)_ = 30.85, *p* < 0.001) was also reflected in increased daytime (*p* < 0.001) and decreased night-time activity levels (*p* < 0.001) in ALAN animals, without changes in the average 24-h locomotor activity (*t*_(14)_ = 1.43, *p* = 0.18; Figures 1B,C).

The RER exhibited daily rhythms (*p* < 0.001; Supplementary Table S2) with distinct higher values during dark/dim periods in comparison to the daytime in both the CTRL and ALAN groups (*F*_(1,14)_ = 369.20, *p* < 0.001; Figures 1D–F; Supplementary Table S2). In the ALAN exposed animals, RER was elevated from ZT5 to ZT7 compared to controls (ZT5: *p* < 0.05, ZT6: *p* < 0.001, ZT7: *p* < 0.001; Figure 1D; Supplementary Table S2), corresponding with increased locomotor activity. Average 24-h RER was not affected by 2 weeks of ALAN exposure (*t*_(14)_ = 0.56, *p* = 0.59; Figure 1F). The RER is determined by the balance between carbohydrate and lipid oxidation throughout the day (Price and Mager, 2020); therefore reflecting substrate oxidation. Overall, ALAN altered the daily rhythms of locomotor activity and RER with an additional peak in locomotor activity in the light phase, as well as a less deep through of the RER.

**FIGURE 2:**
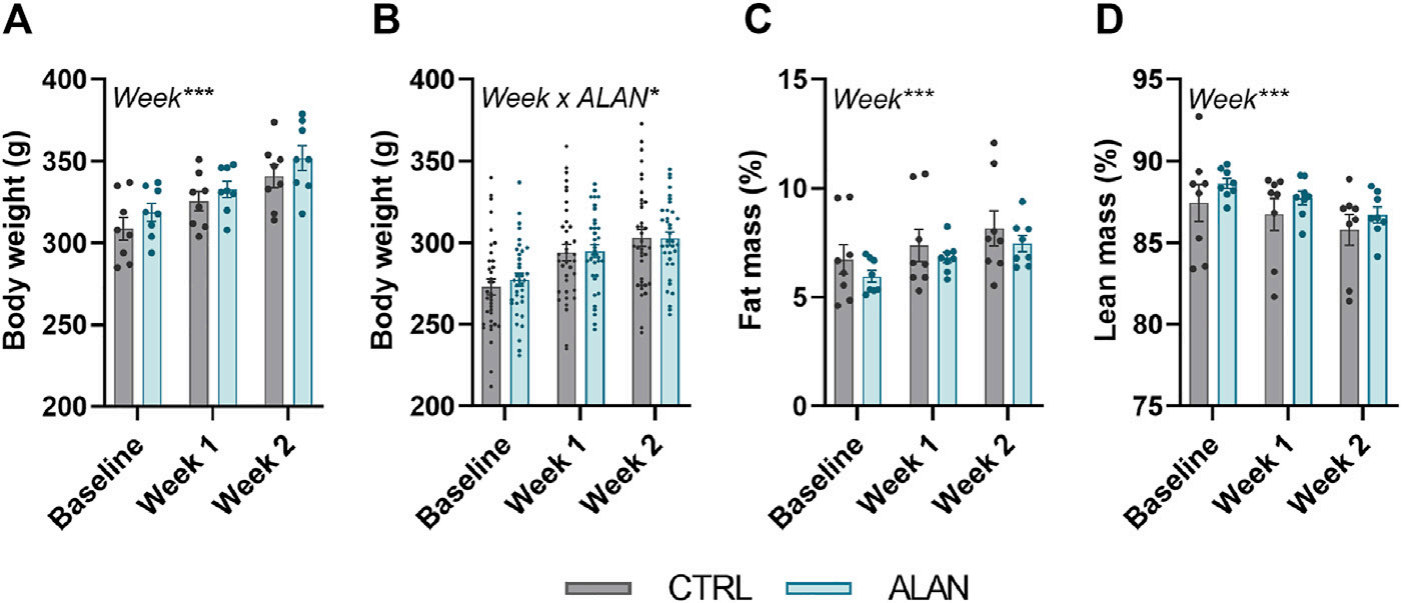
Dim artificial light at night (ALAN) does not affect body weight **(A,B)** and body composition **(C,D)**. Rats were kept either in the standard LD regime (CTRL; grey) or exposed to dim artificial light at night (ALAN; blue) for 2 weeks. Body weight **(A)**, fat **(C)**, and lean mass **(D)** of animals from experiment 1. Body weight of animals from experiment 2 **(B)**. Fat and lean mass were normalized to the body weight. Data represent mean ± SEM with *n* = 8 (experiment 1) and *n* = 36 (experiment 2) per group. Significant effects of week, ALAN or their interaction revealed by two-way ANOVA with repeated measures are indicated by *p < 0.05, and ***p < 0.001.

Body weight (experiment 1: *F*_(2,28)_ = 130.10, experiment 2: *F*_(2,140)_ = 442.3, *p* < 0.001) and fat mass (*F*_(2,28)_ = 38.61, *p* < 0.001) increased and lean mass decreased (*F*_(2,28)_ = 11.21, *p* < 0.001; Figures 2A–D) throughout the 2-week experimental period but did not differ between the CTRL and ALAN group.

### ALAN affects rhythms in glucose metabolism

**FIGURE 3:**
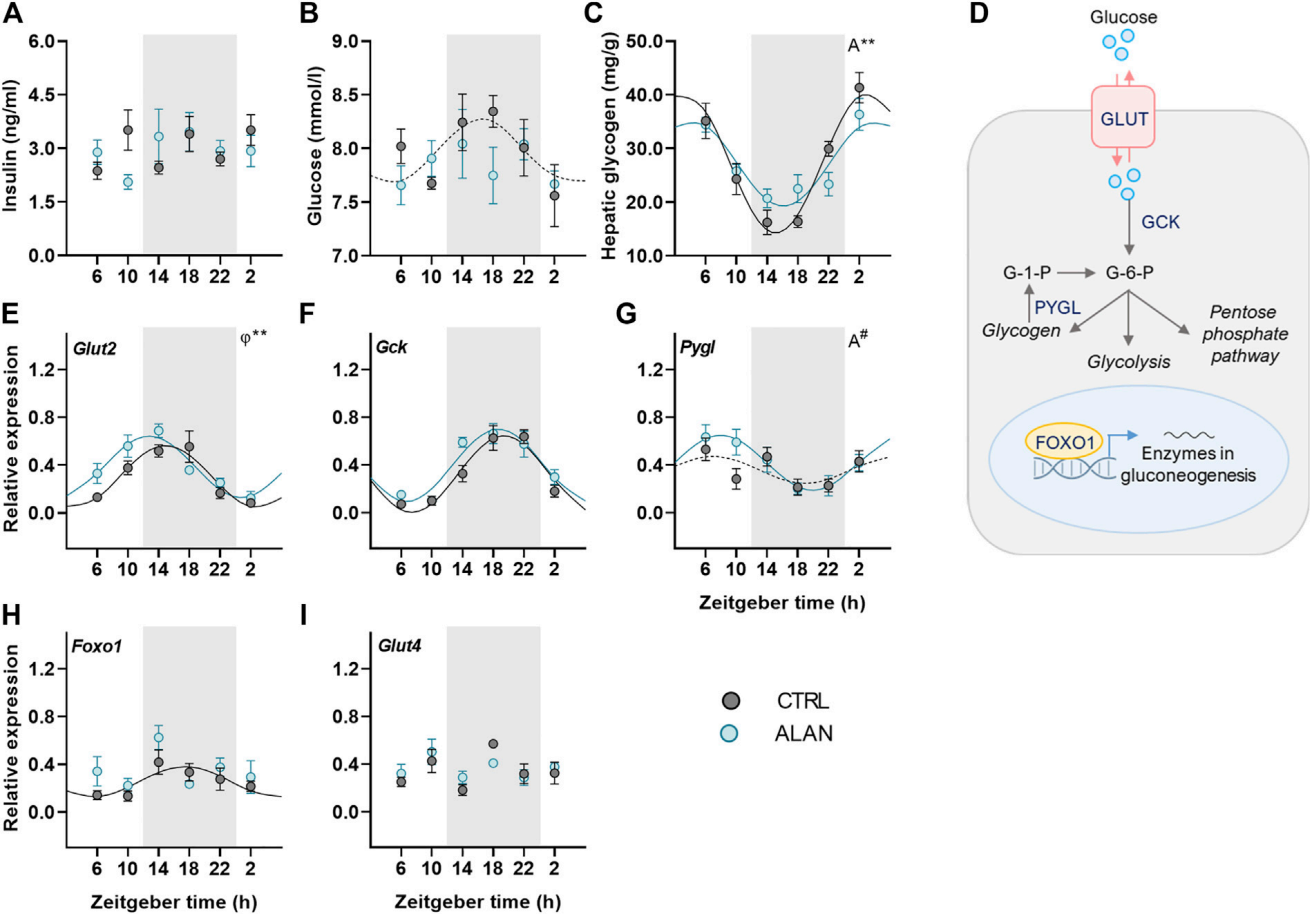
Dim ALAN abolishes the rhythm of glucose and changes the expression of genes involved in glucose uptake and processing. Rats were kept either in the standard LD regime (CTRL; grey) or exposed to dim light at night (ALAN; blue) for 2 weeks. Plasma levels of insulin **(A)** and glucose **(B)**. Hepatic glycogen concentration **(C)**. Scheme of glucose metabolism in the hepatocyte **(D)**. Glucose enters a hepatocyte via glucose transporter 2 (GLUT2) and subsequently is processed by glucokinase (GCK) which results in glucose-6-phosphate (G-6-P). G-6-P can be stored as glycogen or processed in the glycolysis and pentose phosphate pathway. Gluconeogenesis in the liver is regulated by forkhead box protein 1 (FOXO1) which is under the control of metabolic sensors. Glycogen phosphorylase L (the liver form, PYGL) releases glucose-1-phosphate from glycogen stores. Relative mRNA expression of *Glut2*, *Gck*, *Foxo1,* and *Pygl* in the liver **(E–H)**. Relative mRNA levels of *Glut4* in the adipose tissue **(I)**. Grey area indicates the dark/dim light phase. Data represent mean ± SEM with *n* = 5–6 per group. Solid lines indicate the significant daily rhythms (*p* < 0.05) and dashed lines show rhythms with a trend to significance (*p*-value between 0.05 and 0.1). Missing lines represent non-significant 24-h rhythm. Significant changes in amplitude (A) and acrophase (*φ*) revealed by Wald tests are specified by **p < 0.01 and # a trend to significance (*p*-value between 0.05 and 0.1).

To further investigate how ALAN modifies substrate oxidation, we analyzed changes in glucose metabolism. Insulin is a crucial hormone regulating glucose metabolism and lipogenesis in the postprandial state. Plasma insulin levels displayed no daily oscillations (CTRL: *p* = 0.95; ALAN: *p* = 0.31; Figure 3A, Supplementary Table S3) and were not significantly affected by ALAN (*p* = 0.80; Supplementary Table S4). In the CTRL regime, plasma glucose levels tended to be rhythmic (*p* = 0.08) and reached their maximum during the first half of the active phase (Figure 3B, Supplementary Table S3). ALAN exposure abolished this rhythmic pattern (*p* = 0.55). Glucose is stored in the liver and muscles in the form of glycogen. Hepatic glycogen levels showed daily oscillations in both groups (*p* < 0.001), but the amplitude of the rhythm was dampened in the ALAN compared to CTRL group (*p* < 0.01; Figure 3C, Supplementary Table S3).

We also studied the effects of ALAN on rhythms in glucose metabolism on the molecular level (Figure 3D). The expression of hepatic glucose transporter 2 (*Glut2*) was rhythmic in both groups (*p* < 0.001) and ALAN phase-advanced this rhythm by approximately 2 h compared to controls (*p* < 0.01; Figure 3E, Supplementary Table S3). After entering the hepatocyte, glucose is phosphorylated by glucokinase (GCK). The expression of *Gck* mRNA was rhythmic with the peak in the middle of the active phase in both the CTRL and ALAN groups (*p* < 0.001; Figure 3F; Supplementary Table S3) and tended to be higher after the ALAN exposure (*p* = 0.05; Supplementary Table S4). Glucose release from glycogen is regulated by glycogen phosphorylase (PYGL). Hepatic expression of *Pygl* mRNA tended to be rhythmic with the peak in the middle of the light phase in the CTRL group (*p* = 0.09; Supplementary Table S3). ALAN rats showed the daily rhythm of *Pygl* mRNA with tendency to higher amplitude compared to controls (*p* = 0.09; Figure 3G, Supplementary Table S3). Transcription factor, forkhead box protein O1 (FOXO1), which activates expression of genes from the gluconeogenic pathway in a time-of-day dependent manner (Santoleri and Titchenell, 2019), was rhythmic at the mRNA levels with the maximum in the middle of the active phase in the control animals (*p* < 0.05; Supplementary Table S3). This rhythm was suppressed by the ALAN exposure (*p* = 0.54; Figure 3H; Supplementary Table S3). Moreover, *Foxo1* mRNA was up-regulated in the ALAN group as confirmed by two-way ANOVA (*p* < 0.05; Supplementary Table S4). Altogether, these results indicate disturbed glucose uptake and processing in the liver. In the adipose tissue, glucose uptake is mainly facilitated by GLUT4, but its gene expression was arrhythmic in both groups (CTRL: *p* = 0.45; ALAN: *p* = 0.57; Figure 3I; Supplementary Tables S3, S4).

### ALAN affects rhythms in lipid metabolism

**FIGURE 4:**
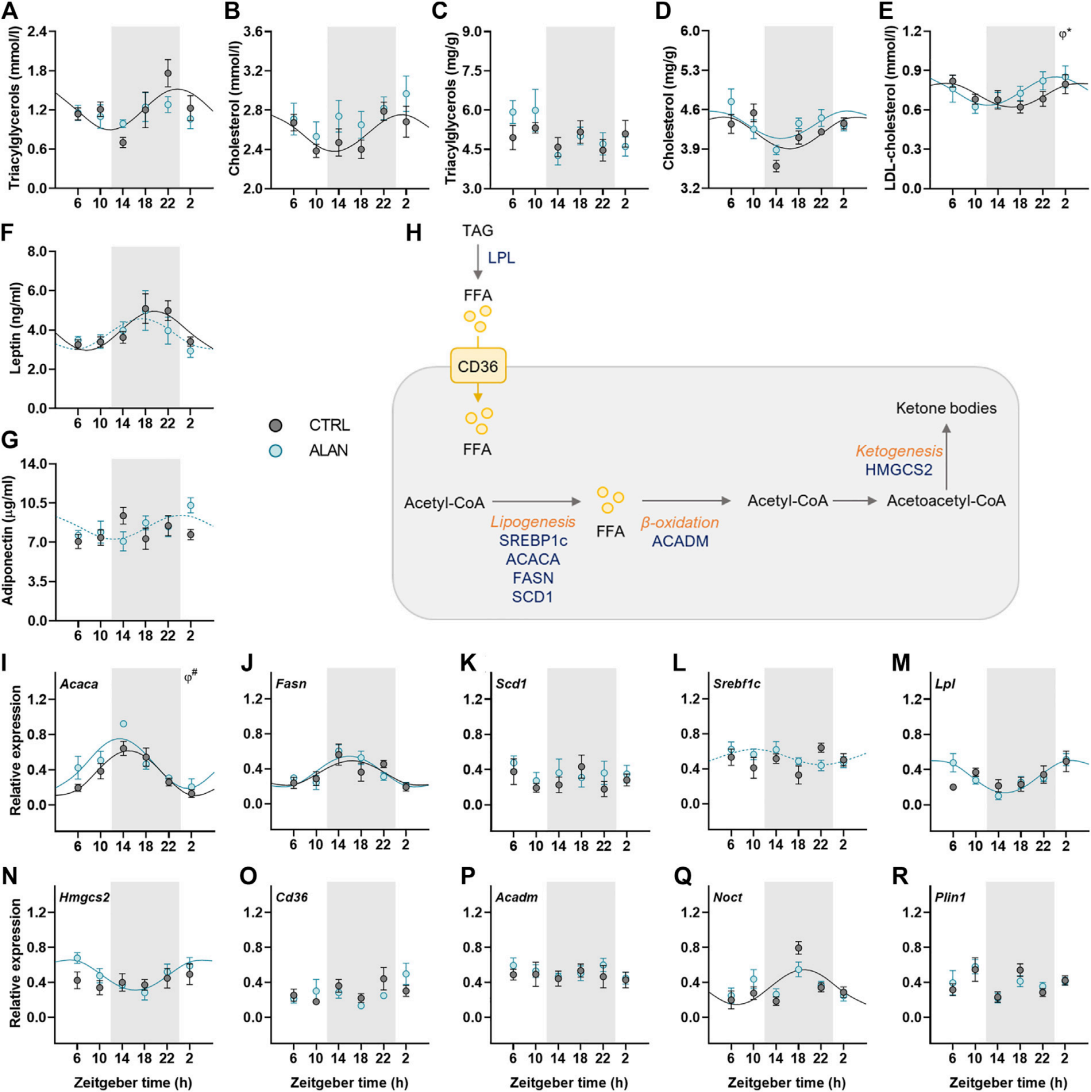
Measured parameters of lipid metabolism respond differently to dim ALAN exposure. Rats were kept either in the standard LD regime (CTRL; grey) or exposed to dim light at night (ALAN; blue) for 2 weeks. The concentration of plasma and hepatic lipids **(A–E)**. Plasma hormone levels of adipokines **(F,G)**. Simplified scheme of measured metabolites and genes in the liver **(H)**. Triacylglycerols are degraded by lipoprotein lipase (LPL) to free fatty acids (FFAs) that can enter the hepatocyte via fatty acid translocase (CD36). FFAs are oxidized to Acetyl-CoA which, in return, acts as a source for lipogenesis regulated by transcription factors (SREBP1C) and lipogenic enzymes (ACACA, FASN, SCD1). Medium-long fatty acid chains are oxidized by ACADM. During fasting, two acetyl-CoA molecules can create acetoacetyl-CoA which is the source for ketone bodies production by enzyme (HMGCS2). Hepatic relative mRNA levels of lipogenic enzymes and transcription factor **(I–L)**, *Lpl* **(M)**, *Hmgcs2* **(N)**, *Cd36* **(O)**, and *Acadm* **(P)**. Relative mRNA expression of *Noct* and *Plin1* in the adipose tissue **(Q,R)**. Grey area indicates the dark/dim light phase. Data represent mean ± SEM with *n* = 5-6 per group. Solid lines indicate the significant daily rhythms (*p* < 0.05) and dashed lines show rhythms with a trend to significance (*p*-value between 0.05 and 0.1). Missing lines represent non-significant 24-h rhythm. Significant changes in acrophase (*φ*) revealed by Wald tests are specified by *p < 0.05 and # a trend to significance (*p*-value between 0.05 and 0.1).

Lipid metabolism is under strong circadian control; therefore, we determined daily rhythms of triacyglycerols and cholesterol, adipokines and expression rhythms of genes involved in lipid metabolism. In CTRL animals, plasma triacylglycerols and cholesterol levels exhibited daily rhythms with their peaks at the end of the dark period and the beginning of the light period, respectively (*p* < 0.05; Figures 4A,B; Supplementary Table S5). This rhythmic pattern was lost after 2 weeks of ALAN exposure (triacylglycerols: *p* = 0.59; cholesterol: *p* = 0.24; Supplementary Table S5). In the liver, triacylglycerol concentrations were arrhythmic in both CTRL (*p* = 0.74) and ALAN groups (*p* = 0.10; Figure 4C; Supplementary Table S5). In contrast, hepatic cholesterol content exhibited a daily rhythm with no significant differences between CTRL (*p* < 0.01) and ALAN animals (*p* < 0.05; Figure 4D; Supplementary Table S5), but two-way ANOVA revealed higher plasma cholesterol levels and a tendency towards higher hepatic cholesterol levels in the ALAN than the CTRL group (*p* = 0.06; Supplementary Table S6). Plasma LDL-cholesterol showed a daily rhythmicity in both groups (CTRL: *p* < 0.05; ALAN: *p* < 0.05), and this rhythm was phase-advanced in the ALAN-exposed animals (*p* < 0.05; Figure 4E; Supplementary Table S5).

Adipokines, such as adiponectin and leptin, regulate food intake and metabolism and their rhythmicity was modified by ALAN. Plasma leptin concentration showed significant daily rhythm in the control animals (*p* < 0.01) which was dampened in the ALAN group (*p* = 0.07; Figure 4F; Supplementary Table S5). Interestingly, arrhythmic adiponectin levels in the control animals (*p* = 0.46) tended to gain rhythmicity after ALAN exposure with the peak at beginning of the light phase (*p* = 0.08; Figure 4G; Supplementary Table S5).

Next, we measured daily profiles of genes involved in fatty acid transport, synthesis, β-oxidation and ketogenesis (Figure 4H). One of the first steps of lipogenesis is catalyzed by acetyl-CoA carboxylase α (ACACA). The rhythmic expression of *Acaca* (*p* < 0.001) tended to be phase-advanced in the liver of ALAN compared to the CTRL rats (*p* = 0.09; Figure 4I; Supplementary Table S5). Moreover, two-way ANOVA revealed higher *Acaca* expression in ALAN than CTRL rats (*p* < 0.05; Supplementary Table S6). Daily gene expression of fatty acid synthase (*Fasn*) was rhythmic in both groups (CTRL: *p* < 0.05; ALAN: *p* < 0.001), whereas, another lipogenic enzyme, stearoyl-CoA desaturase 1 (*Scd1*), did not show a rhythmic pattern (CTRL: *p* = 0.94; ALAN: *p* = 0.78; Figures 4J,K, Supplementary Table S5). Transcription of these lipogenic genes is controlled by the transcription factor sterol regulatory element-binding protein 1c (SREBP1c) whose mRNA was arrhythmic in the CTRL group (*p* = 0.43) but tended to gain rhythmicity under the ALAN conditions (*p* = 0.09; Figure 4L; Supplementary Table S5). Similarly, ALAN-exposed rats showed a rhythmic pattern in the hepatic expression of lipoprotein lipase (*Lpl*, *p* < 0.001) and 3-hydroxy-3-methylglutaryl-CoA synthase 2 (*Hmgcs2*, *p* < 0.01; Figures 4M–N; Supplementary Table S5) with a peak at the beginning of the resting period, whereas no significant rhythms were detected in the CTRL group (*Lpl*: *p* = 0.22; *Hmgcs2*: *p* = 0.54). Daily gene expression of fatty acid transporter (*Cd36*) and acyl-CoA dehydrogenase medium chain (*Acadm*) was arrhythmic in both the CTRL (*Cd36: p* = 0.35, *Acadm: p* = 0.96) and ALAN groups (*Cd36*: *p* = 0.33; *Acadm*: *p* = 0.87; Figures 4O–P; Supplementary Table S5). Nocturnin (*Noct*) is a circadian-regulated protein, which has a role in lipid metabolism, its expression lost the distinct rhythmic pattern in the adipose tissue of the ALAN animals (CTRL: *p* < 0.01; ALAN: *p* = 0.17; Figure 4Q; Supplementary Table S5). However, ALAN did not affect the expression of perilipin1 (*Plin1*) (CTRL: *p* = 0.92; ALAN: *p* = 0.44; Figure 4R; Supplementary Table S5), the enzyme that envelops lipid droplets and prevents lipolysis in adipocytes. Taken together, ALAN profoundly affected control of lipid metabolism. Dim light during the whole night resulted in the loss of rhythmicity in plasma triacyglycerols and cholesterol, as well as *Noct* expression in the adipose tissue, and phase-advanced plasma rhythms of LDL-cholesterol and hepatic *Acaca*. Surprisingly, some parameters (adiponectin, *Lpl*, *Srebf1c*, *Hmgcs2*) even gained rhythmicity.

### Daily rhythms of metabolic sensors were modified by ALAN

**FIGURE 5:**
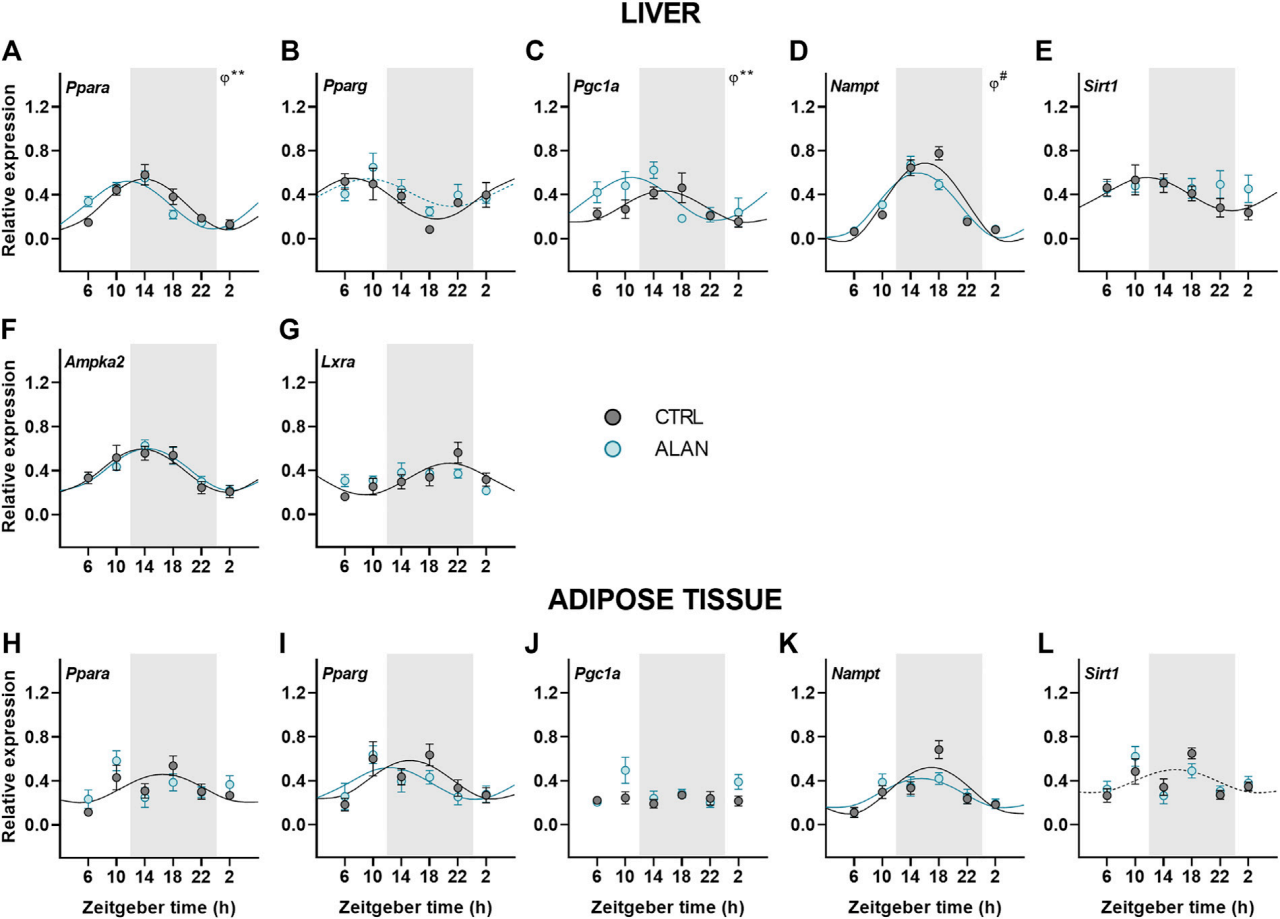
Dim ALAN affected rhythmic expression of metabolic sensors. Rats were kept either in the standard LD regime (CTRL; grey) or exposed to dim light at night (ALAN; blue) for 2 weeks. Relative mRNA levels of metabolic sensors in the liver **(A**–**G)** and adipose tissue **(H–L)**. Grey area indicates the dark/dim light phase. Data represent mean ± SEM with *n* = 5-6 per group. Solid lines indicate the significant daily rhythms (*p* < 0.05) and dashed lines show rhythms with a trend to significance (*p*-value between 0.05 and 0.1). Missing lines represent non-significant 24-h rhythm. Significant changes in acrophase (*φ*) revealed by Wald tests are specified by **p < 0.01 and # a trend to significance (*p*-value between 0.05 and 0.1).

Metabolic sensors play an important role in the interplay between metabolism and the circadian clock. Therefore, we explored how ALAN impacts their daily pattern. Peroxisome proliferator-activated receptors (PPARs) are metabolic transcription factors activated by fatty acids and transcriptionally regulated by the clock (Oishi et al., 2005; Grimaldi et al., 2010). Under the CTRL regime, *Pparα* was rhythmically expressed with the peak during the first half of the active phase in both the liver (*p* < 0.001) and adipose tissue (*p* < 0.05; Figures 5A,H; Supplementary Table S7). Importantly, ALAN advanced the *Pparα* rhythm in the liver (*p* < 0.01) and abolished its rhythmicity in the adipose tissue (*p* = 0.62). On the other hand, ALAN did not affect rhythm of *Pparγ* expression in the liver (CTRL: *p* < 0.01; ALAN: *p* = 0.06) or adipose tissue (*p* < 0.05; Figures 5B,I; Supplementary Table S7). The peroxisome proliferator-activated receptor gamma coactivator 1α (PGC1α), which interacts with PPARs and other transcription factors, participates in the regulation of gluconeogenesis and other metabolic pathways (Puigserver et al., 2003; Wang et al., 2020). In the liver, distinct daily rhythms of *Pgc1a* were found in both CTRL and ALAN groups (*p* < 0.01), but ALAN phase-advanced the acrophase from the middle of the active to the end of the resting phase (*p* < 0.01; Figure 5C; Supplementary Table S7). On the other hand, *Pgc1α* expression in the adipose tissue was arrhythmic in both the CTRL (*p* = 0.89) and ALAN regime (*p* = 0.38; Figure 5J; Supplementary Table S7). Two-way ANOVA revealed a stimulatory effect of ALAN on *Pgc1α* expression in the adipose tissue (*p* < 0.05; Supplementary Table S8). Another important transcription factor and cholesterol sensor, liver X receptor α (LXRα) (Rui, 2014), lost its rhythmic expression in the liver after 2 weeks of ALAN (CTRL: *p* < 0.01; ALAN: *p* = 0.10; Figure 5G; Supplementary Table S7).

Next, we focused on the rhythmic expression of a number of additional genes encoding enzymes responsible for energy metabolism. Hepatic expression of *Nampt* remained rhythmic after ALAN exposure (*p* < 0.001), but the rhythm tended to be phase-advanced in comparison to the CTRL group (*p* = 0.05; Figure 5D; Supplementary Table S7). In the adipose tissue *Nampt* mRNA rhythm was not affected by ALAN (CTRL: *p* < 0.001; ALAN: *p* < 0.01; Figure 5K; Supplementary Table S7). NAMPT is an enzyme involved in NAD^+^ synthesis that regulates SIRT1 activity (Levine et al., 2021); therefore, we also analyzed daily oscillations of *Sirt1*. We found the peak in the first half of the active phase in the liver (*p* < 0.05) and at the light to dark transition in the adipose tissue of CTRL animals, respectively (*p* = 0.09; Figures 5E,L; Supplementary Table S7). Interestingly, *Sirt1* rhythms were abolished in the liver (*p* = 0.93) and adipose tissues (*p* = 0.46) by ALAN exposure. However, protein expression of SIRT1 did not change throughout the day (*F*_(3,40)_ = 1.27, *p* = 0.30) and did not differ between the CTRL and ALAN groups in the liver (*F*_(1,40)_ = 0.59, *p* = 0.45; Supplementary Figure S5E).

An important enzyme controlling energy-producing processes during fasting is AMP-activated proteinkinase (AMPK). Two-way ANOVA revealed that the protein expression of the phosphorylated form of AMPK (pAMPK) was elevated in both the light and dim light period in comparison to the CTRL group (*F*_(1,40)_ = 9.37, *p* < 0.01; Supplementary Figure S5C). However, no significant differences between the ALAN and CTRL groups were found for the unphosphorylated AMPK (*F*_(1,40)_ = 2.27, *p* = 0.14; Supplementary Figure S5B) or the ratio of pAMPK to AMPK (*F*_(1,40)_ = 0.52, *p* = 0.47; Supplementary Figure S5D). The daily rhythm of hepatic *Ampka2* mRNA levels was not affected by ALAN (*p* < 0.001; Figure 5F; Supplementary Table S7). Our results indicate that dim ALAN may disrupt the daily rhythms of some metabolic sensors which could potentially disturb connection between the clockwork and metabolism.

### ALAN effects on the clock gene rhythms in the liver and adipose tissue

**FIGURE 6:**
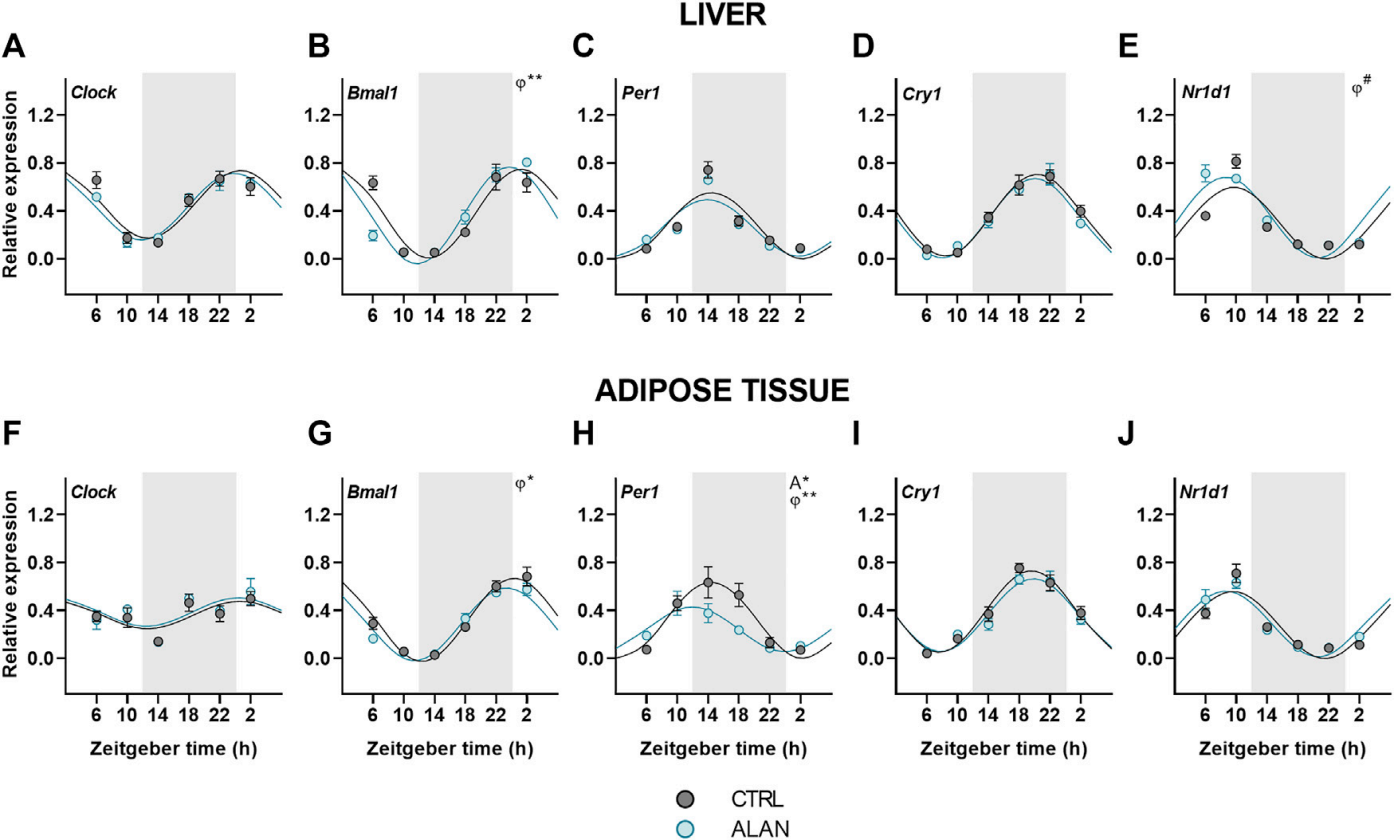
Expression of clock genes remained rhythmic after dim ALAN exposure. Rats were kept either in the standard LD regime (CTRL; grey) or exposed to dim light at night (ALAN; blue) for 2 weeks. Relative mRNA levels of clock genes in the liver **(A–E)** and the adipose tissue **(F–J)**. Grey area indicates the dark/dim light phase. Data represent mean ± SEM with *n* = 5-6 per group. Solid lines indicate the significant daily rhythms (*p* < 0.05) and dashed lines show rhythms with a trend to significance (*p*-value between 0.05 and 0.1). Missing lines represent non-significant 24-h rhythm. Significant changes in amplitude (A) and acrophase (*φ*) revealed by Wald tests are specified by *p < 0.05, **p < 0.01 and # a trend to significance (*p*-value between 0.05 and 0.1).

Finally, we analyzed the effects of ALAN on clock gene rhythms in the liver and adipose tissue. In both tissues, all analyzed clock genes displayed daily rhythmicity in the CTRL and ALAN groups (Figures 6A–J; Supplementary Table S9). The daily rhythm of *Bmal1* expression peaked at the beginning of the resting period in CTRL rats, but its phase was advanced after exposure to ALAN in the liver (*p* < 0.01) and adipose tissue (*p* < 0.05; Figures 6B,G; Supplementary Table S9). *Per1* peaked through the first half of the active period under the CTRL regime in both tissues (*p* < 0.001; Figures 6C,H; Supplementary Table S9). In the adipose tissue, ALAN reduced the amplitude (*p* < 0.05) and phase-advanced the *Per1* rhythm (*p* < 0.01), whereas no changes were observed in the liver (*p* < 0.001; Supplementary Table S9), indicating tissue-specific response to ALAN. Moreover, two-way ANOVA revealed a tendency to lower *Per1* expression in the adipose tissue of ALAN compared to the CTRL animals (*p* = 0.05; Supplementary Table S10). Daily rhythmicity of *Nr1d1* tended to be phase-advanced in the liver after ALAN exposure (*p* = 0.06; Figure 6E; Supplementary Table S9), but was not changed in the adipose tissue (*p* < 0.001; Figure 6J). At the protein level, two-way ANOVA revealed daily rhythms in the hepatic REV-ERBα expression (*F*_(3,40)_ = 3.19, *p* < 0.05), but no effect of ALAN was detected (*F*_(1,40)_ = 0.02, *p* = 0.90; Supplementary Figure S5F). Together, the data show that all investigated clock genes remained rhythmic after ALAN exposure, and only the rhythms of *Bmal1, Per1* and *Nr1d1* showed small phase-advances in a tissue-specific manner.

## Discussion

The increasing rate of nocturnal light pollution calls for a better understanding of its negative effects on metabolism and the uncovering of the mechanisms behind its impact on health. To assess the effects of light pollution on metabolism, we used the model of dim light at night with intensity of ∼2 lx. This intensity is often perceived by humans from outdoor or indoor light sources (Obayashi et al., 2014; Grubisic et al., 2019), but is still higher than normal illumination from moonlight (i.e. 0.1–0.3 lx) (Kyba et al., 2017). Although energy metabolism is under a strong circadian control, data on the daily rhythms of metabolites and metabolic genes after dim ALAN are limited. Therefore, in our study we investigated the daily rhythms in glucose and lipid metabolism together with the daily expression rhythms of key metabolic and clock genes in the liver and adipose tissue as major metabolic sites. We found that ALAN altered daily patterns of locomotor activity and RER, and suppressed or shifted many rhythms in plasma metabolites and metabolic sensors. On the other hand, peripheral clock genes in the liver and adipose tissue retained their robust daily rhythms, although slight changes in acrophases and amplitude were observed. Overall, dim ALAN induced variable changes in the daily patterns of glucose and lipid metabolism suggesting misalignment among different metabolic processes and metabolic tissues.

### Body composition and energy metabolism

In our rat experiment, body and fat mass were not affected by 2-week ALAN exposure, which contrasts with the higher fat mass and body weight in mice reported previously (Fonken et al., 2013; Cissé et al., 2017). The altered body mass in mice was predominantly explained by the increased daytime food intake (Fonken et al., 2010). The daily pattern of food intake was altered also in rats, but without the increased body weight and fat mass (Stenvers et al., 2016; Okuliarova et al., 2020; Okuliarova et al., 2022). These latter results from rats are in line with our current study, and we suggest that this species differences can be caused either by differences in light sensitivity or the study designs, for instance the different photoperiods used (14:10 LD cycle in mice and 12:12 LD cycle in rats).

We found that dim ALAN disrupted the bimodal daily pattern of locomotor activity. As expected, in the control animals, locomotor activity was higher during the dark phase with increased activity at the beginning and the end of the dark phase. Mean 24-h activity was similar in both groups, but in ALAN rats, the expected rise of activity at the onset of the dark phase was dampened. Moreover, dim ALAN increased activity in the light phase, especially at ZT1 and ZT4-5. Altered daily rhythms of activity after dim ALAN were also reported in previous mouse (Fonken et al., 2014) and rat (Stenvers et al., 2016) experiments. In rats, these changes could be partially explained by the appearance of a second rhythm with a period of ∼25 h in addition to the standard 24-h rhythm under dim ALAN conditions (Stenvers et al., 2016). Our findings are different from constant light experiments with higher light intensities when animals became arrhythmic (Polidarová et al., 2011; Stenvers et al., 2016; Báez-Ruiz et al., 2017) or constant dim light conditions in which locomotor activity was either free-running or arrhythmic, depending on the previous exposure to the LD or constant light, respectively (Stenvers et al., 2016). This supports our conclusion that dim light at night provides a condition that is principally different from constant light or constant dim light when animals become free running. With dim light at night, a synchronizing stimulus is still present, albeit its properties are weaker than properties of the standard light:dark cycle.

The observed changes in locomotor activity paralleled the changes in the daily rhythms of food and water intake reported previously (Okuliarova et al., 2022). The elevated daytime food intake was reflected in the RER, which was increased around ZT5-7. The increased RER during the light phase after ALAN exposure reflects a decreased lipid oxidation and increased oxidation of carbohydrates. The reduced utilization of lipids to cover energy needs during the sleep period may lead to an increased accumulation of lipids in the liver (Rumanova et al., 2019; Okuliarova et al., 2020). The modified daily patterns of activity and feeding behavior after dim ALAN exposure can be the consequence of the altered rhythmicity of the central oscillator, as we previously reported (Stenvers et al., 2016; Okuliarova et al., 2022).

### Metabolism

To further understand changes in substrate oxidation after ALAN exposure, we looked at the effects of ALAN on the molecular mechanisms of glucose and lipid metabolism. Plasma glucose lost its rhythmicity and although hepatic glycogen maintained its oscillatory pattern, its amplitude was dampened after ALAN. At the molecular level, we observed a phase shift in the *Glut2* rhythm in the liver of ALAN animals. In line with previous studies (Udoh et al., 2018), control rats showed the peak of *Glut2* rhythm at the beginning of the active period when it is important for the efficient uptake and processing of glucose. It seems that *Glut2* expression is controlled by the molecular clock as daily oscillations of *Glut2* were suppressed in mice with hepatocyte-specific *Bmal1* deletion (Lamia et al., 2008; Ando et al., 2016; Udoh et al., 2018). The phase advance of *Glut2* expression can relate to the shift in *Bmal1* expression in the liver. We assume that under ALAN conditions the uptake of glucose during the daytime is preferentially mediated by hepatic insulin-independent GLUT2 instead of insulin-dependent GLUT4 in the adipose tissue, because its expression was unaffected by ALAN exposure.

In hepatocytes, glucose is phosphorylated by GCK and can enter one of the pathways: glycolysis, pentose pathway or glycogenesis. During the postprandial state, insulin promotes glycogen synthesis in response to the higher glucose levels. Even though we found no changes in plasma insulin levels in ALAN rats, we did find a lower amplitude of the hepatic glycogen rhythm, which could be explained by the increased food intake during the light period and consequently suppressed need for glucose to be released from glycogen stores during the resting phase. Moreover, the change could be promoted by the altered amplitude of *Pygl* and overall higher and arrhythmic *Foxo1* expression in the liver. Glycogen phosphorylase is the rate-limiting enzyme in glycogen catabolism (Agius, 2015) and FOXO1 promotes transcription of gluconeogenic genes (Santoleri and Titchenell, 2019). *Foxo1* is up-regulated in diabetic individuals contributing to hyperglycemia and its inhibition or hepatocyte-specific deletion improves glucose levels (Altomonte et al., 2003; Titchenell et al., 2016). Overall, dim ALAN caused misalignment between rhythms of glucose uptake and processing in the hepatocytes, which in the long-term can negatively affect glucose metabolism.

Lipid metabolism is under strong circadian control. Plasma triacylglycerols and cholesterol lost their rhythmic patterns, and LDL-cholesterol was phase-advanced under the ALAN regime. Moreover, blood and hepatic cholesterol levels were higher in ALAN compared to control rats. In our previous study, we found an increased hepatic lipid content in rats exposed to ALAN (Okuliarova et al., 2020) and this lipid accumulation was even more pronounced when spontaneously hypertensive rats, which are genetically insulin resistant, were exposed to dim ALAN (Rumanova et al., 2019). In the current study, *Srebf1c* gained rhythmicity with the peak around ZT10, a few hours after the increased daytime food intake. SREBP1c is a transcription factor promoting *de novo* lipogenesis and such a gain of rhythmicity in *Srebf1c* and *Lpl* was also observed when rats were fed six meals a day (De Goede et al., 2018). It seems that in conditions with an attenuated central oscillator functioning the timing of food intake can contribute to the gain of rhythmicity in some genes, because this effect was also found in animals on a high-fat diet (Dyar et al., 2018) or with a disrupted SCN (Kolbe et al., 2016).

The transcription factors SREBP1c and LXRα stimulate syntheses of fatty acids and regulate cholesterol transport (Rui, 2014). Thus, changed rhythmicity of these factors can contribute to the increased plasma and hepatic cholesterol concentrations, which could be further promoted by the gain of rhythmicity in plasma adiponectin.

### Metabolic sensors

Metabolism and molecular clocks are mutually inter-connected through metabolic sensors that are regulated by both and ensure fine-tuning of the circadian clock to the metabolic status of an organism. In addition to clock and clock-controlled genes, metabolic sensors can be considered as the accessory elements of the molecular clock. In the liver, ALAN exposure resulted in the arrhythmicity of *Sirt1* and *Lxrα*, while *Nampt, Pparα* and *Pgc1α* were phase-advanced. In the adipose tissue, *Sirt1* and *Pparα* became arrhythmic. The loss of rhythmicity and altered daily pattern of these sensors could disrupt the circadian control of metabolism. Sirtuin one adjusts the molecular clock by deacetylation of PER2 and BMAL1 (Nakahata et al., 2008; Levine et al., 2020). In both the liver and adipose tissue, the rhythmic profile of *Sirt1* was lost after ALAN, indicating an important pathway via which light pollution can interfere with metabolic control. The oxidized form of NAD acts as the cofactor for SIRT1, whereas NADH inhibits SIRT1 to help energy conservation during the early hours of the resting phase (Levine et al., 2021). Indeed, we found low *Sirt1* values at the beginning of the resting phase in the control rats. The transcriptional control of *Sirt1* is poorly understood. One of the assumed mechanisms is the regulation via PPARγ:SIRT1 complex (Han et al., 2010); therefore, the misbalanced metabolic state could be one of the factors contributing to the loss of *Sirt1* rhythmicity. NAMPT is an important enzyme in the NAD^+^ salvage pathway (Reinke and Asher, 2019; Van Der Spek et al., 2021). ALAN rats showed a tendency towards a phase-advanced *Nampt* rhythm, which could be explained by the shift of *Bmal1* expression since the heterodimer CLOCK:BMAL1 promotes its transcription (Lee and Kim, 2013). Another indirect regulatory mechanism of *Nampt* is via AMPK through CRY degradation, affecting the heterodimer CLOCK:BMAL1 (Lee and Kim, 2013).

PPARs represent an important connection between the circadian clock and metabolism and are regulated by the clock (Oishi et al., 2005; Grimaldi et al., 2010), the availability of fatty acids (Botta et al., 2018) and SIRT1 (Kosgei et al., 2020). While PPARα controls energy producing processes, PPARγ stimulates lipogenesis, especially in the adipose tissue. Rhythms of *Pparγ* were not affected by ALAN exposure in neither the liver nor adipose tissue. On the other hand, the *Pparα* rhythm was phase-advanced in the liver and completely lost in the adipose tissue. The daily rhythm of the transcriptional coactivator *Pgc1α*, which interacts with both PPARs, was also phase-advanced in the liver and unchanged in the adipose tissue after ALAN. PPARs, together with PGC1α, promote *Bmal1* transcription via PPAR-response element of the promoter (Oishi et al., 2005; Kriebs et al., 2017) and transcription of *Pparα* and *Pgc1α* is regulated by CLOCK:BMAL1 complex. PPARα stimulates energy production via the promotion of the transcription of genes encoding enzymes involved in the β-oxidation of fatty acids and ketogenesis in the liver. In the adipose tissue, PPARα controls fatty acids oxidation and improves insulin sensitivity (Goto et al., 2011; Takahashi et al., 2017). Therefore, its disrupted rhythmicity can interfere with insulin signaling. Since PPARα and SIRT1 are known for the stimulation of energy producing processes, the suppression of their rhythmicity or a phase advance could disturb necessary links between the liver and adipose tissue, compromising the ability of adipose tissue to store excess of lipids.

*Pparγ* expression was unaffected on the mRNA level, but its protein nuclear translocation is facilitated by nocturnin in the adipose tissue (Kawai et al., 2010), and *Noct* expression became arrhythmic. It is likely that the misalignment between gene expression rhythms of metabolic sensors in the different tissues will have a negative effect on metabolic homeostasis and the balance between the molecular clock and metabolism.

### Peripheral clocks

Despite the attenuated rhythmicity of the central oscillator and its hormonal outputs after exposure to dim ALAN, the daily oscillations of clock genes in the liver and adipose tissue were largely preserved with only small changes in their acrophases or amplitudes (*Bmal1, Per1, Nr1d1*). Our data are in line with other models with a disturbed central oscillator, also showing maintained daily clock gene rhythms in the liver and adipose tissue, which were often phase-advanced (Husse et al., 2014; Kolbe et al., 2016; Kolbe et al., 2019; Van Der Spek et al., 2021). This phenomenon is observed only under the LD regime and not in constant darkness, suggesting an important role for behavioral and endocrine rhythms in the synchronization of peripheral clocks. Our previous study using the same lighting conditions (Okuliarova et al., 2022) demonstrated that the daily rhythm of food intake was preserved, but attenuated and the corticosterone rhythm was phase-advanced after ALAN exposure. Both rhythms are well-known to contribute to the rhythmicity of peripheral clocks. Indeed, corticosterone is known to adjust the phase of daily rhythms in peripheral tissues (Balsalobre et al., 2000; Pezük et al., 2012) and also food intake represents a strong Zeitgeber for peripheral clocks, especially the liver clock, independent of the LD cycle (Damiola et al., 2000; Oosterman et al., 2020). Thus, the attenuated circadian signaling of the central clock due to ALAN is sufficient for peripheral clocks to keep ticking, but they are not able anymore to synchronize all rhythmic metabolic processes in the different organs due to their different sensitivities to the conflicting information from the environmental light/dark and behavioral sleep/wake rhythms.

## Conclusion

In conclusion, here we provide evidence that even low-intensity nocturnal light can affect daily rhythms of metabolic pathways. Despite the attenuated central oscillator and its hormonal and behavioral outputs, the peripheral clocks maintained their daily oscillations with only slight changes in acrophases and amplitudes. However, the rhythmic expression of metabolic sensors was vastly different after ALAN exposure, probably the result of the arrhythmic metabolite levels. Thus, even small changes in the daily pattern of behavior and energy metabolism are sufficient to disturb daily rhythms of substrate oxidation and the metabolic status of organism. Disrupted or misaligned rhythms of metabolism are often observed in metabolic diseases, such as diabetes or obesity, chronodisruption by ALAN exposure could, thus, be one of the triggers to accelerate their development. For instance, the inappropriate timing can potentially interfere with storage of fatty acids in the adipose tissue and increase lipid accumulation in the liver. In the long term, the lipid accumulation can progress into the non-alcoholic fatty liver disease especially in combination with other risk factors.

## Data Availability

The raw data supporting the conclusion of this article will be made available by the authors, without undue reservation.
