# Supplementary material for: Exposure to dim light at night alters daily rhythms of glucose and lipid metabolism in rats
Source: Front Physiol. 2022 Aug 29;13:973461. doi: 10.3389/fphys.2022.973461 (PMC9465160; doi:10.3389/fphys.2022.973461)
Supplement: Supplementary file 1 [file Image1.pdf]

frontiers

Supplementary Material

Supplementary Figures

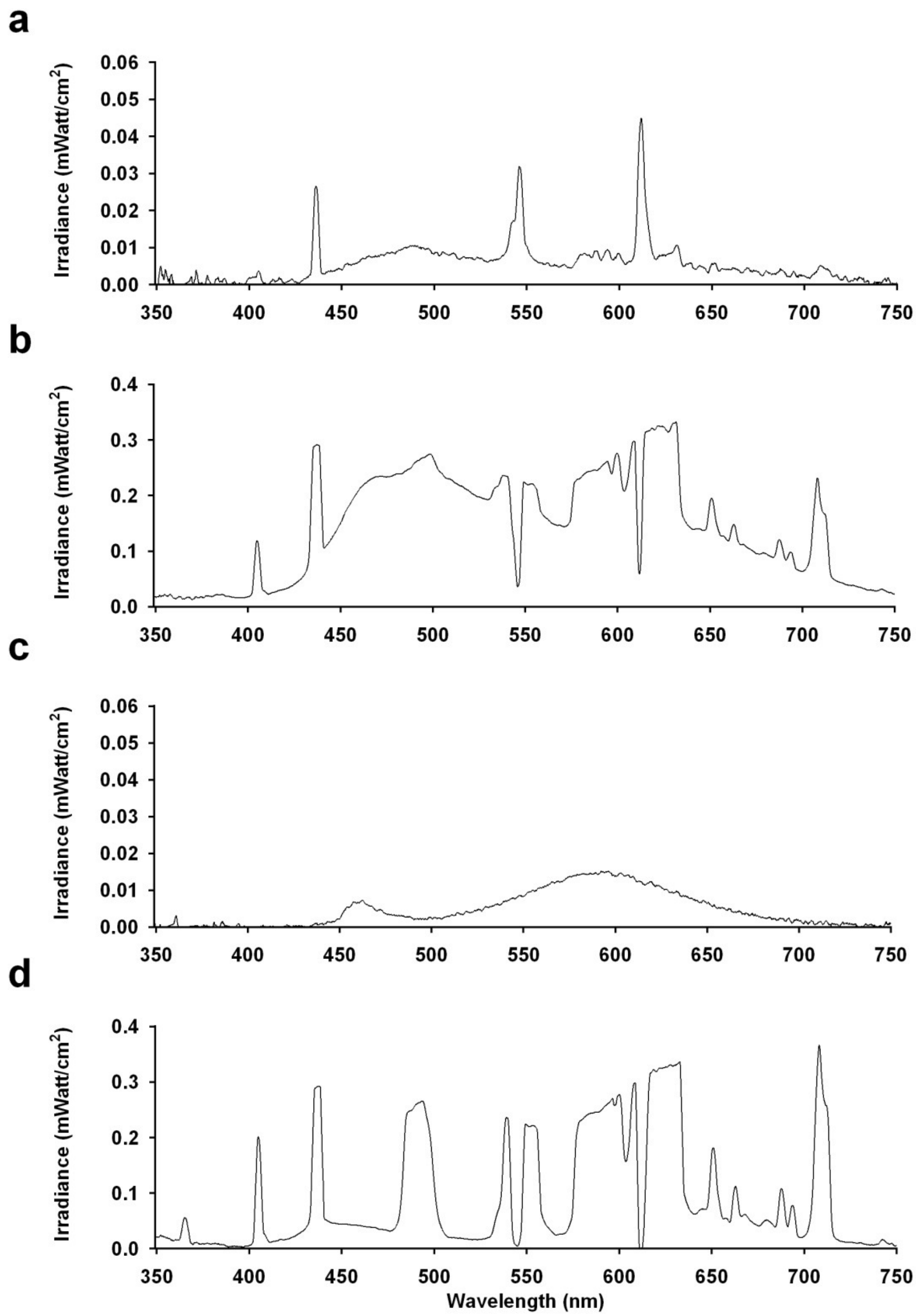

Figure S1. Spectral power distributions of light sources used in Experiment 1. Dim light (5 lux) emitted by white LED lights (**c**) and bright light (150-200 lux) (**d**) emitted by white fluorescent tubes for Experiment 1. Note the 10-fold difference in y-axis units between dim and bright light. Spectral

power distributions were measured with an AvaSpec 2048-SPU (Avantus BV, the Netherlands) light meter. The figure is an abstract from the figure in Stenvers et al., 2016 published in Scientific Reports by Nature Portfolio (Stenvers et al., 2016).

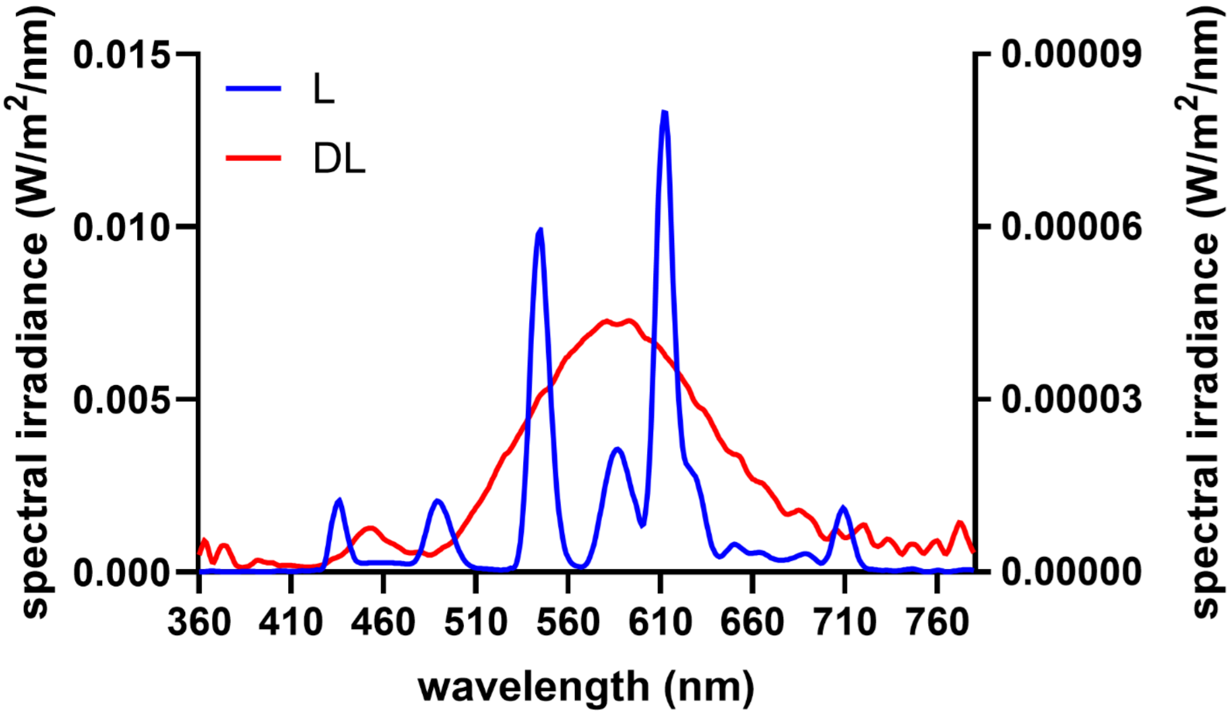

Figure S2. Spectral power distributions of light sources used in Experiment 2. Bright white light (150–200 lx, L) used during the light phase (blue, left y axis) and dim light (~2 lx, DL) during the night phase (red, right y axis). Spectral power distribution was measured with an illuminance spectrophotometer CL-500A (Konica Minolta Sensing Europe BV, Germany)

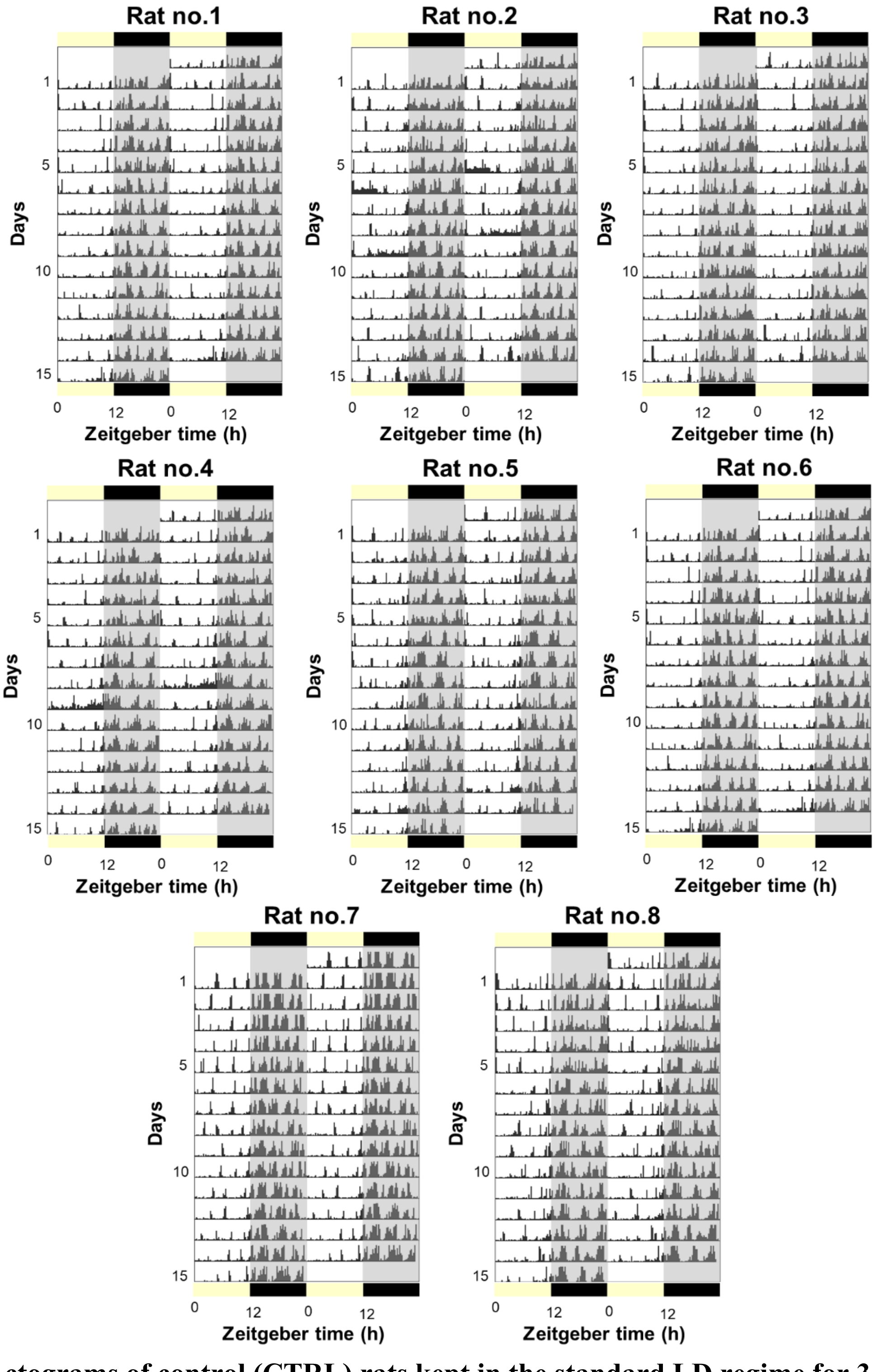

Figure S3. Actograms of control (CTRL) rats kept in the standard LD regime for 3 weeks (Experiment 1). Five days per week are shown in the actogram: baseline week – day 1 to 5, week 1 – day 6 to 10, week 2 – day 11 to 15. Each week, two days were skipped because of the weekly cleaning of the cages. Horizontal bars at the top and bottom of actograms indicate light (light yellow) and dark phase (black).

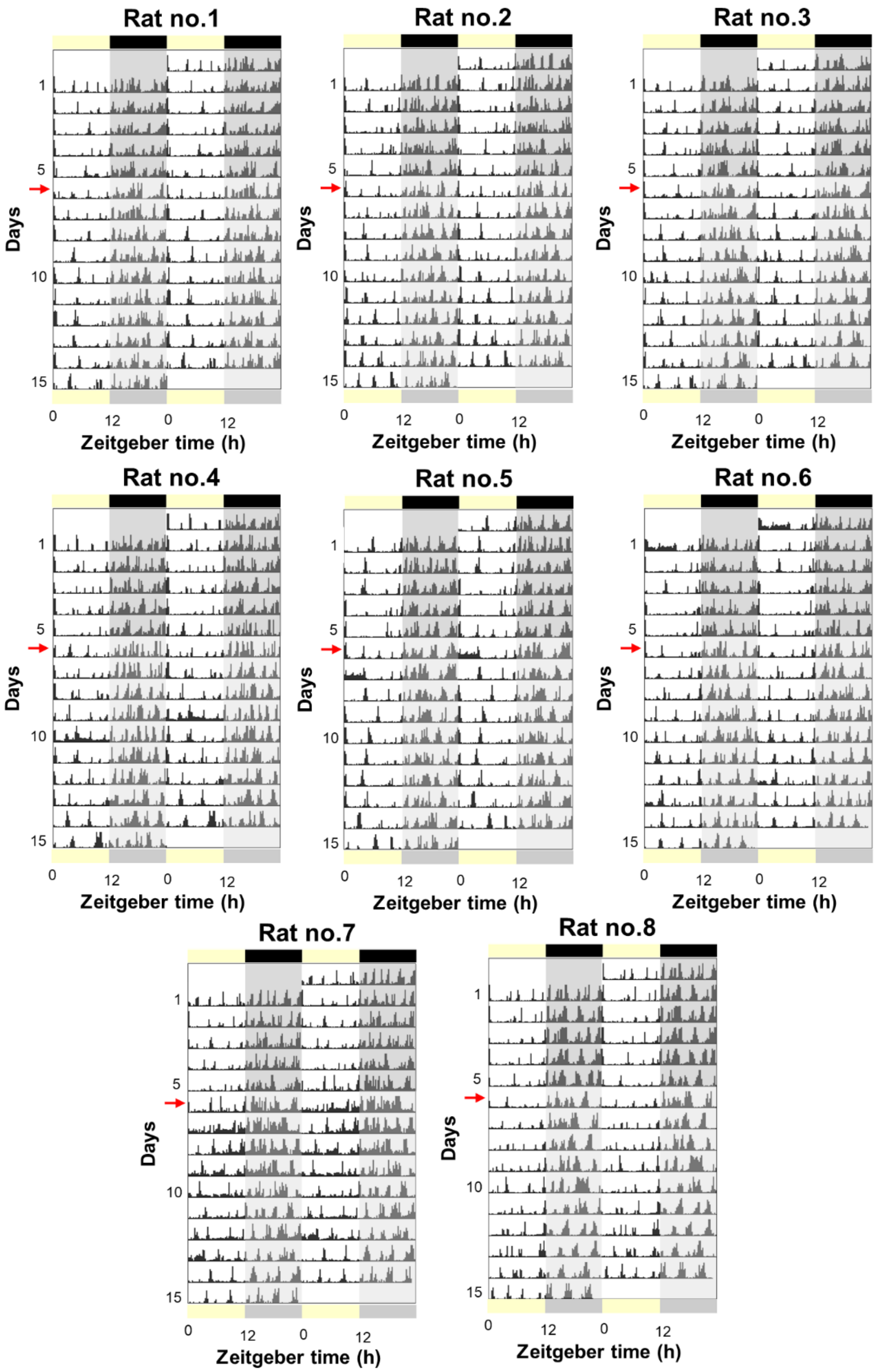

Figure S4. **Actograms of rats exposed to dim light at night (ALAN) for 2 weeks (Experiment 1).** During the baseline weeks animals were kept in the standard LD regime and after, animals were exposed to dim light at night (the change in light regimes is indicated by red arrow). Five days per week are shown in the actogram: baseline week – day 1 to 5, week 1 – day 6 to 10, week 2 – day 11 to 15. Each week, two days were skipped because of the weekly cleaning of the cages. Horizontal bars at the top and bottom of actograms indicate light (light yellow), dark (black) and dim light phase (grey).

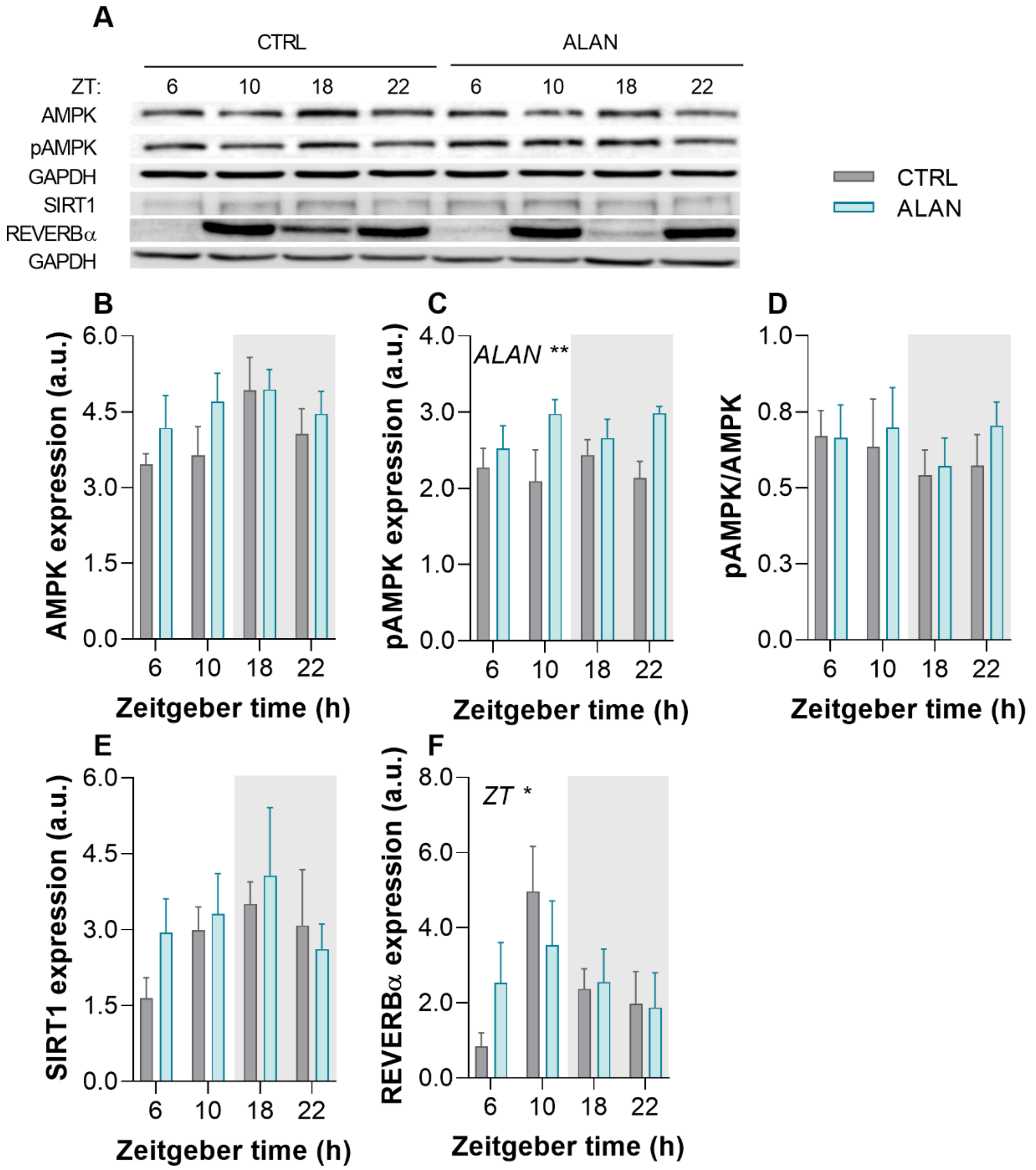

Figure S5. Protein expression of metabolic sensors was unaffected by dim ALAN except for phosphorylated AMP-activated protein kinase. Rats were kept either in the standard LD regime (CTRL; grey) or exposed to the dim light at night (ALAN; blue) for 2 weeks. Representative blots of AMPK, pAMPK, SIRT1 and REVERBα normalized to housekeeping protein GAPDH (shown below target proteins) with ZT representing zeitgeber time (**A**). Relative protein expression of unphosphorylated (**B**) and phosphorylated (**C**) AMPK. Ratio of pAMPK to AMPK (**D**). Relative protein expression of SIRT1 (**E**) and clock protein REVERBα (**F**). Grey area indicates the dark/dim light phase. Data represent mean ± SEM with n=5-6 per group. Significant changes in factors (ZT, ALAN) or their interaction revealed by two-way ANOVA are specified by * $p < 0.05$, ** $p < 0.01$.

Supplementary Tables

Table S1. Primer sequences for real-time PCR.

Gene	Accession number	Forward primer	Reverse primer
Reference genes			
B2m	NM_012512.2	5'- TCGTGCTTGCCATTCAGAAAACT -3'	5'- AGCAGTTGAGGAAGTTGGGCT -3'
Rn18s	NR_046237	5'- GTAACCCGTTGAACCCCATT -3'	5'- CCATCCAATCGGTAGTAGCG -3'
Target genes			
Acaca	NM_022193.1	5'- GCAGTCTCCCAACTCCTACG -3'	5'- CAGTCCACCATCACTCAGCC -3'
Acadm	NM_016986.2	5'- GGAGCCGGGACTAGGGTTTA -3'	5'- CTGGCAAACTTCCGAGCAAT -3'
Ampka2	NM_023991.1	5'- GACAGGCCATAAAGTGGCAG -3'	5'- AGTGCTGATCACTTGGTAGAGTTT -3'
Bmal1	NM_024362.2	5'- CACCTTGCGGAATGTCACAG -3'	5'- TACTTCCTTGGTCCACGGGT -3'
Cd36	NM_031561	5'- CGGCGATGAGAAAGCAGAAA -3'	5'- GGCTCATCCACTACTTATTTTCC -3'
Clock	NM_021856.2	5'- CTTACAGACATCTCGGTTGCTC -3'	5'- TCTGAGGAAGCGTGTGCTAC -3'
Cry1	NM_198750.2	5'- GTGTGGAAGTCATCGTGCG -3'	5'- ACGTCTGATGTGATGGTGTCT -3'
Fasn	NM_017332.1	5'- GAGTCTGTCTCCCGCTTGAC -3'	5'- TTGCCTTGCTCACCTTCGAG -3'
Foxo1	NM_001191846.2	5'- CAGCAAATCAAGTTATGGAGGA -3'	5'- TATCATTGTGGGGAGGAGAGTC -3'
Gck	NM_001270849.1	5'- GGAGTATGACCGGATGGTGG -3'	5'- GCCTCTCCGTGGAACAGAAG -3'
Glut2	NM_012879.2	5'- GAAGGATCAAAGCCATGTTGG -3'	5'- CCTGATACGCTTCTTCCAGCA -3'
Glut4	NM_012751.1	5'- TATGTTGCGGATGCTATGGGT -3'	5'- AATGTCCGGCCTCTGGTTTC -3'
Hmgcs2	NM_173094.2	5'- ACAAGTCCAAGGCTGTCAAG -3'	5'- CATAGCGACCATCCCAGTAG -3'
Lpl	NM_012598.2	5'- TCATCAACTGGTTGGAGGAAG -3'	5'- ACGAAATCCGCATCATCAGG -3'
Lxra	NM_031627.2	5'- TGCCTGATGTTTCTCCTGACTCTG -3'	5'- AGTTGATTGGGGCATCCTGGC -3'
Nampt	NM_177928.3	5'- AAGTTGCTGCCACCTTACCTT -3'	5'- TTCATTCCCTCGACAATCTCCT -3'
Noct	NM_138526.1	5'- CAGTAGCCATCCTCCCATTAG -3'	5'- ATTTCCTCTCCTCCCATTTGA -3'
Nr1d1	NM_145775.2	5'- TCCCACATACTTCCCACCATCA -3'	5'- CACTCGGCTGCTGTCTTCCAT -3'
Per1	NM_001034125.1	5'- ATCGTCCATGTGGGAGACAAG -3'	5'- TTGCTCTTCCTTCTGGGTGTG -3'
Pgc1a	NM_031347.1	5'- AACGATGACCCTCCTCACAC -3'	5'- GTTGTTGGTTTGGCTTGAGCA -3'
Plin1	NM_001308145.1	5'- GTACACTATGTCCCGCTTCC -3'	5'- CCACCTCTGCTGGAGGATTA -3'
Ppara	NM_013196.1	5'- GACTAGCAACAATCCGCCTT -3'	5'- GAAGAATCGGACCTCTGCCT -3'
Pparg	NM_001145367.1	5'- TCCAAGAATACCAAAGTGCGA -3'	5'- CCATGAGGGAGTTTGAAGGC -3'
Pygl	NM_022268.1	5'- ATCCACTCGGACATCGTGAA -3'	5'- CCTGGGTTGCAGAGTAAGAG -3'
Scd1	NM_139192.2	5'- AAACCTGCAGAATGGACGAGA -3'	5'- GGGTCGTGGATATCTTCTCTCA -3'
Sirt1	NM_001372090.1	5'- TTATGCTCGCCTTGCTGTGG -3'	5'- CTGTCCGGGATATATTTCCTTTGC -3'
Srebf1c	NM_001276707.1	5'- GCCATCGACTACATCCGCTT -3'	5'- CCAGGTCTTTCAGTGATTTGCT -3'

Abbreviations: *Acaca* - acetyl-CoA carboxylase α, *Acadm* - acyl-CoA dehydrogenase medium chain, *Ampka2* - protein kinase AMP-activated catalytic subunit α 2, *B2m* – β-2-microglobulin, *Bmal1* - brain and muscle ARNT-like 1, *Cd36* - cluster of differentiation 36 (fatty acid translocase), *Clock* - circadian locomotor output cycles kaput, *Cry1* – cryptochrome 1, *Fasn* – fatty acid synthase, *Foxo1* – forkhead box protein O1, *Gck* – glucokinase, *Glut2* – glucose transporter 2, *Glut4* – glucose transporter 4, *Hmgcs2* - 3-hydroxy-3-methylglutaryl-CoA synthase 2, *Lpl* – lipoprotein lipase, *Lxra* – liver X receptor α, *Nampt* - nicotinamide phosphoribosyltransferase, *Noct* – nocturnin, *Nr1d1* - nuclear receptor subfamily 1 group d member 1 (REV-ERBα), *Per1* – period 1, *Pgc1a* - peroxisome proliferator-activated receptor γ coactivator 1α, *Plin1* – perilipin 1, *Ppara* - peroxisome proliferator-activated receptor α, *Pparg* - peroxisome proliferator-activated receptor γ, *Pygl* – glycogen phosphorylase L (liver form), *Scd1* - stearoyl-CoA desaturase 1, *Sirt1* – sirtuin 1, *Srepf1c* - sterol regulatory element binding transcription factor 1, *Rn18s* - 18S ribosomal RNA

Table S2. Cosinor analysis and the two-way ANOVA with repeated measures of locomotor activity and respiratory exchange ratio in rats kept in either the control regime (CTRL) or exposed to dim light at night (ALAN).

	Cosinor analysis						Two-way ANOVA with RM		
	**Mesor**	**Amplitude**	**Acrophase (hh:mm)**	**R^2**	***F*-test**	***p*-value**	**ZT**	**ALAN**	**Interaction**
**Locomotor activity (counts)**									
CTRL	358.03 ± 33.33	263.91 ± 47.13	17:09 ± 00:41	0.62	$F_{(2,189)} = 60.22$	***p* < 0.001**	$F_{(23,322)} = 22.20$	$F_{(1,322)} = 2.04$	$F_{(23,322)} = 5.68$
ALAN	333.66 ± 25.39	185.36 ± 35.92**	18:57 ± 00:44	0.59	$F_{(2,189)} = 51.17$	***p* < 0.001**	***p* < 0.001**	p = 0.176	***p* < 0.001**
**RER**									
CTRL	0.987 ± 0.003	0.048 ± 0.005	17:55 ± 00:23	0.82	$F_{(2,189)} = 196.36$	***p* < 0.001**	$F_{(23,322)} = 75.24$	$F_{(1,322)} = 0.30$	$F_{(23,322)} = 2.94$
ALAN	0.991 ± 0.003	0.039 ± 0.004**	18:28 ± 00:24	0.81	$F_{(2,189)} = 174.00$	***p* < 0.001**	***p* < 0.001**	p = 0.594	***p* < 0.001**

Cosinor analysis:
Rhythm parameters: mesor ± 95% confidence limits (the time series mean), amplitude ± 95% confidence limits (one-half the peak-to-trough difference) and acrophase ± 95% confidence limits (the peak of the fitted curve). R^2, *F*- and *p*-values describe the significance of 24-h rhythms. A rhythm with $p < 0.05$ is considered significant and with a trend to significance if *p*-value is between 0.05 and 0.1, both are indicated with bold font.

Two-way ANOVA with repeated measures:
F- and *P*-values of factors Zeitgeber time (ZT), ALAN and their interaction. Significant effects are indicated with bold font.

Abbrevations: RER – respiratory exchange ratio

Table S3. The cosinor analysis of genes involved in glucose metabolism of rats kept in either the control regime (CTRL) or exposed to dim light at night (ALAN).

	Mesor	Amplitude	Acrophase (hh:mm)	R^2	*F*-test	*p*-value
**PLASMA**						
**Insulin (ng/ml)**						
CTRL	-	-	-	-	$F_{(2,33)} = 0.05$	p = 0.95
ALAN	-	-	-	-	$F_{(2,33)} = 1.22$	p = 0.31
**Glucose (mmol/l)**						
CTRL	7.98 ± 0.18	0.30 ± 0.25	16:38 ± 03:12	0.38	$F_{(2,33)} = 2.74$	**p = 0.08**
ALAN	-	-	-	-	$F_{(2,33)} = 0.61$	p = 0.55
**LIVER**						
**Glycogen (mg/g)**						
CTRL	27.28 ± 1.86	13.07 ± 2.64	03:09 ± 00:46	0.86	$F_{(2,32)} = 47.13$	**p < 0.001**
ALAN	27.20 ± 1.78	7.96 ± 2.52**	04:06 ± 01:12	0.73	$F_{(2,33)} = 19.21$	**p < 0.001**
Foxo1						
CTRL	1.24 ± 0.11	0.23 ± 0.15	17:27 ± 02:25	0.49	$F_{(2,32)} = 4.51$	**p < 0.05**
ALAN	-	-	-	-	$F_{(2,33)} = 0.64$	p = 0.54
Gck						
CTRL	0.87 ± 0.11	0.69 ± 0.16	19:24 ± 00:53	0.83	$F_{(2,32)} = 35.84$	**p < 0.001**
ALAN	1.03 ± 0.12	0.65 ± 0.17	18:40 ± 00:58	0.80	$F_{(2,33)} = 29.58$	**p < 0.001**
Glut2						
CTRL	1.23 ± 0.13	0.63 ± 0.19	14:55 ± 01:07	0.76	$F_{(2,33)} = 22.17$	**p < 0.001**
ALAN	1.42 ± 0.13	0.63 ± 0.18	12:47 ± 01:04**	0.77	$F_{(2,33)} = 24.31$	**p < 0.001**
Pygl						
CTRL	1.10 ± 0.08	0.13 ± 0.12	06:55 ± 03:19	0.37	$F_{(2,33)} = 2.55$	**p = 0.09**
ALAN	1.17 ± 0.08	0.28 ± 0.12#	07:56 ± 01:34	0.63	$F_{(2,33)} = 10.97$	**p < 0.001**
**EPIDIDYMAL FAT**						
Glut4						
CTRL	-	-	-	-	$F_{(2,32)} = 0.82$	p = 0.45
ALAN	-	-	-	-	$F_{(2,32)} = 0.56$	p = 0.57

Rhythm parameters: mesor ± 95% confidence limits (the time series mean), amplitude ± 95% confidence limits (one-half the peak-to-trough difference) and acrophase ± 95% confidence limits (the peak of the fitted curve). R^2, *F*- and *p*-values describe the significance of 24-h rhythms. A rhythm with $p < 0.05$ is considered significant and with a trend to significance if *p*-value is between 0.05 and 0.1, both are indicated with bold font.

Abbreviations: *Foxo1* – forkhead box protein O1, *Gck* – glucokinase, *Glut2* – glucose transporter 2, *Glut4* – glucose transporter 4, *Pygl* – glycogen phosphorylase L (liver form)

Table S4. The two-way ANOVA of genes involved in glucose metabolism of rats kept in either the control regime (CTRL) or exposed to dim light at night (ALAN).

	Time	Regime	Interaction
**PLASMA**			
**Insulin (ng/ml)**	$F_{(5,60)}$ = 0.99<br>p = 0.43	$F_{(1,60)}$ = 0.06<br>p = 0.80	$F_{(5,60)}$ = 1.93<br>p = 0.10
**Glucose (mmol/l)**	$F_{(5,60)}$ = 1.70<br>p = 0.15	$F_{(1,60)}$ = 1.11<br>p = 0.30	$F_{(5,60)}$ = 1.08<br>p = 0.38
**LIVER**			
**Glycogen (mg/g)**	$F_{(5,59)}$ = 26.25<br>p < 0.001 ***	$F_{(1,59)}$ = 0.00<br>p = 0.99	$F_{(5,59)}$ = 2.58<br>p <0.05 *
Foxo1	$F_{(5,59)}$ = 4.04<br>p < 0.01 **	$F_{(1,59)}$ = 4.06<br>p < 0.05 *	$F_{(5,59)}$ = 0.83<br>p = 0.53
Gck	$F_{(5,59)}$ = 27.79<br>p < 0.001 ***	$F_{(1,59)}$ = 3.88<br>p = 0.05 #	$F_{(5,59)}$ = 1.51<br>p = 0.20
Glut2	$F_{(5,60)}$ = 34.61<br>p < 0.001 ***	$F_{(1,60)}$ = 1.02<br>p = 0.32	$F_{(5,60)}$ = 0.89<br>p = 0.49
Pygl	$F_{(5,60)}$ = 5.86<br>p < 0.001 ***	$F_{(1,60)}$ = 1.54<br>p = 0.22	$F_{(5,60)}$ = 1.16<br>p = 0.34
**EPIDIDYMAL FAT**			
Glut4	$F_{(5,58)}$ = 4.25<br>p < 0.01 **	$F_{(1,58)}$ = 0.18<br>p = 0.68	$F_{(5,58)}$ = 1.03<br>p = 0.41

F- and *P*-values of factors time, regime and their interaction. Significant effects are indicated by * $p < 0.05$, ** $p < 0.01$, *** $p < 0.001$ and # a trend to significance (p-value between 0.05 and 0.1). Abbreviations: *Foxo1* – forkhead box protein O1, *Gck* – glucokinase, *Glut2* – glucose transporter 2, *Glut4* – glucose transporter 4, *Pygl* – glycogen phosphorylase L (liver form)

Table S5. The cosinor analysis of genes involved in lipid metabolism of rats kept in either the control regime (CTRL) or exposed to dim light at night (ALAN).

	Mesor	Amplitude	Acrophase (hh:mm)	R^2	*F*-test	*p*-value
**PLASMA**						
**Adiponectin (μg/ml)**						
CTRL	-	-	-	-	$F_{(2,33)} = 0.80$	p = 0.46
ALAN	8.35 ± 0.63	1.07 ± 0.89	00:17 ± 03:01	0.38	$F_{(2,33)} = 2.78$	**p = 0.08**
**Cholesterol (mmol/l)**						
CTRL	2.57 ± 0.09	0.19 ± 0.13	01:13 ± 02:32	0.46	$F_{(2,33)} = 4.35$	**p < 0.05**
ALAN	-	-	-	-	$F_{(2,33)} = 1.49$	p = 0.24
**LDL-cholesterol (mmol/l)**						
CTRL	0.71 ± 0.04	0.09 ± 0.06	04:31 ± 02:31	0.46	$F_{(2,33)} = 4.40$	**p < 0.05**
ALAN	0.74 ± 0.06	0.11 ± 0.08	00:17 ± 02:46*	0.43	$F_{(2,33)} = 3.68$	**p < 0.05**
**Leptin (ng/ml)**						
CTRL	3.96 ± 0.34	1.00 ± 0.49	19:34 ± 01:52	0.57	$F_{(2,33)} = 8.02$	**p < 0.01**
ALAN	3.80 ± 0.45	0.78 ± 0.64	17:19 ± 03:07	0.39	$F_{(2,33)} = 2.89$	**p = 0.07**
**Triacylglycerols (mmol/l)**						
CTRL	1.21 ± 0.15	0.31 ± 0.21	23:41 ± 02:37	0.45	$F_{(2,33)} = 4.09$	**p < 0.05**
ALAN	-	-	-	-	$F_{(2,33)} = 0.54$	p = 0.59
**LIVER**						
**Hepatic cholesterol (mg/g)**						
CTRL	4.19 ± 0.12	0.29 ± 0.17	04:22 ± 02:15	0.50	$F_{(2,33)} = 5.56$	**p < 0.01**
ALAN	4.33 ± 0.13	0.25 ± 0.18	02:51 ± 02:49	0.43	$F_{(2,32)} = 3.70$	**p < 0.05**
**Hepatic triacylglycerols (mg/g)**						
CTRL	-	-	-	-	$F_{(2,33)} = 0.31$	p = 0.74
ALAN	-	-	-	-	$F_{(2,32)} = 2.43$	p = 0.10
Acaca						
CTRL	1.13 ± 0.11	0.52 ± 0.16	14:57 ± 01:09	0.75	$F_{(2,33)} = 21.14$	**p < 0.001**
ALAN	1.36 ± 0.15	0.57 ± 0.21	13:22 ± 01:23#	0.69	$F_{(2,33)} = 14.62$	**p < 0.001**
Acadm						
CTRL	-	-	-	-	$F_{(2,33)} = 0.04$	p = 0.96
ALAN	-	-	-	-	$F_{(2,33)} = 0.12$	p = 0.87
Cd36						
CTRL	-	-	-	-	$F_{(2,32)} = 1.10$	p = 0.35
ALAN	-	-	-	-	$F_{(2,32)} = 1.15$	p = 0.33
Fasn						
CTRL	1.00 ± 0.13	0.27 ± 0.18	16:23 ± 02:33	0.45	$F_{(2,33)} = 4.29$	**p < 0.05**
ALAN	1.03 ± 0.10	0.34 ± 0.14	15:54 ± 01:34	0.64	$F_{(2,33)} = 11.36$	**p < 0.001**
Hmgcs2						
CTRL	-	-	-	-	$F_{(2,33)} = 0.63$	p = 0.54
ALAN	1.00 ± 0.04	0.12 ± 0.06	04:19 ± 01:51	0.58	$F_{(2,33)} = 8.24$	**p < 0.01**
Lpl						
CTRL	-	-	-	-	$F_{(2,32)} = 1.59$	p = 0.22
ALAN	0.97 ± 0.12	0.44 ± 0.17	03:11 ± 01:29	0.66	$F_{(2,33)} = 12.60$	**p < 0.001**
Scd1						
CTRL	-	-	-	-	$F_{(2,32)} = 0.06$	p = 0.94
ALAN	-	-	-	-	$F_{(2,30)} = 0.26$	p = 0.78
Srebf1c						
CTRL	-	-	-	-	$F_{(2,33)} = 0.86$	p = 0.43
ALAN	1.14 ± 0.09	0.14 ± 0.12	10:06 ± 03:18	0.37	$F_{(2,33)} = 2.59$	**p = 0.09**
**EPIDIDYMAL FAT**						
Noct						
CTRL	8.83 ± 1.09	2.93 ± 1.56	18:56 ± 01:59	0.54	$F_{(2,32)} = 6.75$	**p < 0.01**
ALAN	-	-	-	-	$F_{(2,33)} = 1.88$	p = 0.17
Plin1						
CTRL	-	-	-	-	$F_{(2,32)} = 0.09$	p = 0.92
ALAN	-	-	-	-	$F_{(2,33)} = 0.85$	p = 0.44

Rhythm parameters: mesor ± 95% confidence limits (the time series mean), amplitude ± 95% confidence limits (one-half the peak-to-trough difference) and acrophase ± 95% confidence limits (the peak of the fitted curve). R^2, *F*- and *p*-values describe the significance of 24-h rhythms. A rhythm with $p < 0.05$ is considered significant and with a trend to significance if *p*-value is between 0.05 and 0.1, both are indicated with bold font.
Abbreviations: *Acaca* - acetyl-CoA carboxylase α, *Acadm* - acyl-CoA dehydrogenase medium chain, *Cd36* - cluster of differentiation 36 (fatty acid translocase), *Fasn* – fatty acid synthase, *Hmgcs2* - 3-hydroxy-3-methylglutaryl-CoA synthase 2, *Lpl* – lipoprotein lipase, *Noct* – nocturnin, *Plin1* – perilipin 1, *Scd1* - stearoyl-CoA desaturase 1, *Srepf1c* - sterol regulatory element binding transcription factor 1

Table S6. The two-way ANOVA of genes involved in lipid metabolism of rats kept in either the control regime (CTRL) or exposed to dim light at night (ALAN).

	Time	Regime	Interaction
	**PLASMA**		
**Adiponectin (µg/ml)**	$F_{(5,60)}$ = 1.17<br>p = 0.33	$F_{(1,60)}$ = 1.06<br>p = 0.31	$F_{(5,60)}$ = 2.31<br>p = 0.06 #
**Cholesterol (mmol/l)**	$F_{(5,60)}$ = 2.50<br>p < 0.05 *	$F_{(1,60)}$ = 4.95<br>p < 0.05 *	$F_{(5,60)}$ = 0.38<br>p = 0.86
**LDL-cholesterol (mmol/l)**	$F_{(5,60)}$ = 2.42<br>p < 0.05 *	$F_{(1,60)}$ = 0.66<br>p = 0.42	$F_{(5,60)}$ = 0.84<br>p = 0.53
**Leptin (ng/ml)**	$F_{(5,60)}$ = 4.16<br>p < 0.01 **	$F_{(1,60)}$ = 0.64<br>p = 0.43	$F_{(5,60)}$ = 0.69<br>p = 0.63
**Triacylglycerols (mmol/l)**	$F_{(5,60)}$ = 3.46<br>p < 0.01 **	$F_{(1,60)}$ = 0.24<br>p = 0.63	$F_{(5,60)}$ = 1.25<br>p = 0.30
	**LIVER**		
**Hepatic cholesterol (mg/g)**	$F_{(5,59)}$ = 8.71<br>p <0.001 ***	$F_{(1,59)}$ = 3.72<br>p = 0.06 #	$F_{(5,59)}$ = 1.77<br>p = 0.13
**Hepatic triacylglycerols (mg/g)**	$F_{(5,59)}$ = 2.33<br>p = 0.054 #	$F_{(1,59)}$ = 0.35<br>p = 0.56	$F_{(5,59)}$ = 0.90<br>p = 0.49
Acaca	$F_{(5,60)}$ = 15.75<br>p < 0.001 ***	$F_{(1,60)}$ = 6.42<br>p < 0.05 *	$F_{(5,60)}$ = 1.41<br>p = 0.23
Acadm	$F_{(5,60)}$ = 0.49<br>p = 0.780	$F_{(1,60)}$ = 0.90<br>p = 0.35	$F_{(5,60)}$ = 0.23<br>p = 0.95
Cd36	$F_{(5,58)}$ = 2.00<br>p = 0.092 #	$F_{(1,58)}$ = 0.09<br>p = 0.77	$F_{(5,58)}$ = 1.56<br>p = 0.19
Fasn	$F_{(5,60)}$ = 7.68<br>p < 0.001 ***	$F_{(1,60)}$ = 0.15<br>p = 0.70	$F_{(5,60)}$ = 1.03<br>p = 0.41
Lpl	$F_{(5,59)}$ = 5.01<br>p < 0.001 ***	$F_{(1,59)}$ = 0.05<br>p = 0.83	$F_{(5,59)}$ = 1.76<br>p = 0.14
Hmgcs2	$F_{(5,60)}$ = 2.12<br>p = 0.075 #	$F_{(1,60)}$ = 2.18<br>p = 0.15	$F_{(5,60)}$ = 0.93<br>p = 0.47
Scd1	$F_{(5,56)}$ = 0.90<br>p = 0.488	$F_{(1,56)}$ = 1.43<br>p = 0.24	$F_{(5,56)}$ = 0.23<br>p = 0.81
Srebf1c	$F_{(5,60)}$ = 1.30<br>p = 0.277	$F_{(1,60)}$ = 1.03<br>p = 0.32	$F_{(5,60)}$ = 1.60<br>p = 0.17
	**EPIDIDYMAL FAT**		
Noct	$F_{(5,59)}$ = 10.53<br>p < 0.001 ***	$F_{(1,59)}$ = 0.01<br>p = 0.91	$F_{(5,59)}$ = 1.81<br>p = 0.12
Plin1	$F_{(5,59)}$ = 4.62<br>p < 0.01 **	$F_{(1,59)}$ = 0.01<br>p = 0.93	$F_{(5,59)}$ = 0.46<br>p = 0.81

F- and *P*-values of factors time, regime and their interaction. Significant effects are indicated by * $p < 0.05$, ** $p < 0.01$, *** $p < 0.001$ and # a trend to significance (p-value between 0.05 and 0.1). Abbreviations: *Acaca* - acetyl-CoA carboxylase α, *Acadm* - acyl-CoA dehydrogenase medium chain, *Cd36* - cluster of differentiation 36 (fatty acid translocase), *Fasn* – fatty acid synthase, *Hmgcs2* - 3-hydroxy-3-methylglutaryl-CoA synthase 2, *Lpl* – lipoprotein lipase, *Noct* – nocturnin, *Plin1* – perilipin 1, *Scd1* - stearoyl-CoA desaturase 1, *Srepf1c* - sterol regulatory element binding transcription factor 1

Table S7. The cosinor analysis of metabolic sensors in rats kept in either the control regime (CTRL) or exposed to dim light at night (ALAN).

	Mesor	Amplitude	Acrophase (hh:mm)	R^2	*F*-test	*p*-value
			**LIVER**			
Ampkα2						
CTRL	0.93 ± 0.07	0.24 ± 0.10	13:36 ± 01:34	0.64	$F_{(2,33)}$ = 11.45	**p < 0.001**
ALAN	0.94 ± 0.05	0.23 ± 0.07	14:23 ± 01:07	0.76	$F_{(2,33)}$ = 22.67	**p < 0.001**
Nampt						
CTRL	1.26 ± 0.17	1.30 ± 0.24	16:02 ± 00:42	0.88	$F_{(2,33)}$ = 55.96	**p < 0.001**
ALAN	1.18 ± 0.13	1.06 ± 0.18	15:04 ± 00:40#	0.89	$F_{(2,33)}$ = 63.96	**p < 0.001**
Lxrα						
CTRL	1.00 ± 0.06	0.15 ± 0.09	20:46 ± 02:11	0.51	$F_{(2,33)}$ = 5.83	**p < 0.01**
ALAN	-	-	-	-	$F_{(2,33)}$ = 2.52	p = 0.10
Pparα						
CTRL	1.15 ± 0.11	0.55 ± 0.15	13:54 ± 01:02	0.78	$F_{(2,33)}$ = 25.99	**p < 0.001**
ALAN	1.14 ± 0.09	0.52 ± 0.12	11:39 ± 00:54**	0.82	$F_{(2,33)}$ = 34.64	**p < 0.001**
Pparγ						
CTRL	0.97 ± 0.10	0.25 ± 0.14	06:58 ± 02:07	0.55	$F_{(2,31)}$ = 6.56	**p < 0.01**
ALAN	1.04 ± 0.09	0.17 ± 0.13	09:17 ± 02:59	0.40	$F_{(2,33)}$ = 3.13	**p = 0.06**
Pgc1α						
CTRL	1.27 ± 0.13	0.33 ± 0.19	15:21 ± 02:13	0.51	$F_{(2,33)}$ = 5.71	**p < 0.01**
ALAN	1.43 ± 0.17	0.44 ± 0.24	10:49 ± 02:06**	0.53	$F_{(2,33)}$ = 6.34	**p < 0.01**
Sirt1						
CTRL	1.05 ± 0.06	0.14 ± 0.09	11:37 ± 02:28	0.47	$F_{(2,33)}$ = 4.62	**p < 0.05**
ALAN	-	-	-	-	$F_{(2,33)}$ = 0.07	p = 0.93
			**EPIDIDYMAL FAT**			
Nampt						
CTRL	5.60 ± 0.69	2.70 ± 0.99	16:54 ± 01:22	0.69	$F_{(2,32)}$ = 14.40	**p < 0.001**
ALAN	5.33 ± 0.65	1.71 ± 0.92	15:29 ± 02:03	0.54	$F_{(2,33)}$ = 6.68	**p < 0.01**
Pparα						
CTRL	8.81 ± 0.98	1.98 ± 1.40	16:32 ± 02:39	0.44	$F_{(2,32)}$ = 3.84	**p < 0.05**
ALAN	-	-	-	-	$F_{(2,33)}$ = 0.48	p = 0.62
Pparγ						
CTRL	8.73 ± 0.87	1.99 ± 1.22	15:15 ± 02:24	0.50	$F_{(2,31)}$ = 5.13	**p < 0.05**
ALAN	8.37 ± 0.81	1.62 ± 1.15	12:53 ± 02:42	0.43	$F_{(2,33)}$ = 3.82	**p < 0.05**
Pgc1α						
CTRL	-	-	-	-	$F_{(2,32)}$ = 0.11	p = 0.89
ALAN	-	-	-	-	$F_{(2,33)}$ = 1.01	p = 0.38
Sirt1						
CTRL	8.34 ± 0.81	1.33 ± 1.15	15:44 ± 03:17	0.37	$F_{(2,32)}$ = 2.55	**p = 0.09**
ALAN	-	-	-	-	$F_{(2,33)}$ = 0.80	p = 0.46

Rhythm parameters: mesor ± 95% confidence limits (the time series mean), amplitude ± 95% confidence limits (one-half the peak-to-trough difference) and acrophase ± 95% confidence limits (the peak of the fitted curve). R^2, *F*- and *p*-values describe the significance of 24-h rhythms. A rhythm with $p < 0.05$ is considered significant and with a trend to significance if *p*-value is between 0.05 and 0.1, both are indicated with bold font.

Abbrevations: *Ampka2* - protein kinase AMP-activated catalytic subunit α 2, *Lxra* – liver X receptor α, *Nampt* - nicotinamide phosphoribosyltransferase, *Pgc1a* - peroxisome proliferator-activated receptor γ coactivator 1α, *Ppara* - peroxisome proliferator-activated receptor α, *Pparg* - peroxisome proliferator-activated receptor γ, *Sirt1* – sirtuin 1

Table S8. The two-way ANOVA of metabolic sensors in rats kept in either the control regime (CTRL) or exposed to dim light at night (ALAN).

	Time	Regime	Interaction
**LIVER**			
Ampkα2	$F_{(5,60)} = 11.83$<br>$p < 0.001$ ***	$F_{(1,60)} = 0.05$<br>$p = 0.82$	$F_{(5,60)} = 0.38$<br>$p = 0.86$
Lxrα	$F_{(5,60)} = 3.62$<br>$p < 0.01$ **	$F_{(1,60)} = 0.01$<br>$p = 0.91$	$F_{(5,60)} = 2.07$<br>$p = 0.08$ #
Nampt	$F_{(5,60)} = 85.68$<br>$p < 0.001$ ***	$F_{(1,60)} = 0.95$<br>$p = 0.33$	$F_{(5,60)} = 5.25$<br>$p < 0.001$ ***
Pparα	$F_{(5,60)} = 23.50$<br>$p < 0.001$ ***	$F_{(1,60)} = 0.05$<br>$p = 0.91$	$F_{(5,60)} = 2.61$<br>$p < 0.05$ *
Pparγ	$F_{(5,58)} = 4.94$<br>$p < 0.001$ ***	$F_{(1,58)} = 0.92$<br>$p = 0.34$	$F_{(5,58)} = 0.78$<br>$p = 0.57$
Pgc1α	$F_{(5,60)} = 3.82$<br>$p < 0.01$ **	$F_{(1,60)} = 2.16$<br>$p = 0.15$	$F_{(5,60)} = 2.49$<br>$p < 0.05$ *
Sirt1	$F_{(5,60)} = 1.06$<br>$p = 0.393$	$F_{(1,60)} = 1.67$<br>$p = 0.20$	$F_{(5,60)} = 0.85$<br>$p = 0.52$
**EPIDIDYMAL FAT**			
Nampt	$F_{(5,59)} = 13.36$<br>$p < 0.001$ ***	$F_{(1,59)} = 0.43$<br>$p = 0.51$	$F_{(5,59)} = 2.18$<br>$p = 0.07$ #
Pparα	$F_{(5,59)} = 4.98$<br><0.001 ***	$F_{(1,59)} = 0.29$<br>$p = 0.59$	$F_{(5,59)} = 1.18$<br>$p = 0.33$
Pparγ	$F_{(5,58)} = 6.14$<br>$p <0.001$ ***	$F_{(1,58)} = 0.52$<br>$p = 0.47$	$F_{(5,58)} = 0.63$<br>$p = 0.68$
Pgc1α	$F_{(5,59)} = 2.31$<br>$p = 0.06$ #	$F_{(1,59)} = 4.74$<br>$p < 0.05$ *	$F_{(5,59)} = 1.93$<br>$p = 0.10$
Sirt1	$F_{(5,59)} = 7.20$<br>$p <0.001$ ***	$F_{(1,59)} = 0.01$<br>$p = 0.93$	$F_{(5,59)} = 1.17$<br>$p = 0.33$

F- and *P*-values of factors time, regime and their interaction. Significant effects are indicated by * $p < 0.05$, ** $p < 0.01$, *** $p < 0.001$ and # a trend to significance (*p*-value between 0.05 and 0.1). Abbrevations: *Ampka2* - protein kinase AMP-activated catalytic subunit α 2, *Lxra* – liver X receptor α, *Nampt* - nicotinamide phosphoribosyltransferase, *Pgc1a* - peroxisome proliferator-activated receptor γ coactivator 1α, *Ppara* - peroxisome proliferator-activated receptor α, *Pparg* - peroxisome proliferator-activated receptor γ, *Sirt1* – sirtuin 1

Table S9. The cosinor analysis of clock genes in rats kept in either the control regime (CTRL) or exposed to dim light at night (ALAN).

	Mesor	Amplitude	Acrophase (hh:mm)	R^2	*F*-test	*p*-value
			**LIVER**			
Bmal1						
CTRL	0.99 ± 0.16	1.10 ± 0.23	01:17 ± 00:48	0.86	$F_{(2,32)} = 45.33$	p < 0.001
ALAN	0.96 ± 0.12	1.20 ± 0.17	23:46 ± 00:33**	0.93	$F_{(2,32)} = 98.27$	p < 0.001
Clock						
CTRL	1.23 ± 0.09	0.47 ± 0.13	00:41 ± 01:02	0.78	$F_{(2,33)} = 26.26$	p < 0.001
ALAN	1.20 ± 0.07	0.46 ± 0.09	23:53 ± 00:45	0.86	$F_{(2,33)} = 48.77$	p < 0.001
Cry1						
CTRL	1.12 ± 0.10	0.93 ± 0.14	20:22 ± 00:34	0.92	$F_{(2,32)} = 84.26$	p < 0.001
ALAN	1.05 ± 0.11	0.91 ± 0.15	20:01 ± 00:38	0.90	$F_{(2,33)} = 68.22$	p < 0.001
Nr1d1						
CTRL	0.90 ± 0.23	1.43 ± 0.33	09:40 ± 00:53	0.83	$F_{(2,33)} = 36.43$	p < 0.001
ALAN	1.12 ± 0.19	1.61 ± 0.27	08:37 ± 00:39#	0.90	$F_{(2,33)} = 67.08$	p < 0.001
Per1						
CTRL	1.43 ± 0.31	1.85 ± 0.44	14:29 ± 00:55	0.82	$F_{(2,33)} = 33.51$	p < 0.001
ALAN	1.31 ± 0.25	1.58 ± 0.35	13:57 ± 00:51	0.84	$F_{(2,33)} = 38.39$	p < 0.001
			**EPIDIDYMAL FAT**			
Bmal1						
CTRL	8.52 ± 0.93	9.14 ± 1.30	00:21 ± 00:34	0.92	$F_{(2,32)} = 94.57$	p < 0.001
ALAN	7.54 ± 0.93	8.00 ± 1.09	23:23 ± 00:31*	0.93	$F_{(2,33)} = 101.80$	p < 0.001
Clock						
CTRL	8.78 ± 0.89	1.83 ± 1.24	00:36 ± 02:40	0.45	$F_{(2,32)} = 4.17$	p < 0.05
ALAN	9.17 ± 0.95	1.90 ± 1.34	00:25 ± 02:43	0.43	$F_{(2,33)} = 3.82$	p < 0.05
Cry1						
CTRL	8.37 ± 0.80	6.64 ± 1.14	19:35 ± 00:39	0.90	$F_{(2,32)} = 65.27$	p < 0.001
ALAN	7.78 ± 0.82	5.92 ± 1.16	19:50 ± 00:45	0.87	$F_{(2,33)} = 49.94$	p < 0.001
Nr1d1						
CTRL	9.50 ± 2.06	13.13 ± 2.90	09:31 ± 00:51	0.84	$F_{(2,32)} = 39.31$	p < 0.001
ALAN	9.93 ± 1.86	12.82 ± 2.63	08:36 ± 00:47	0.86	$F_{(2,32)} = 45.54$	p < 0.001
Per1						
CTRL	5.80 ± 0.92	4.87 ± 1.30	14:25 ± 01:02	0.79	$F_{(2,32)} = 27.00$	p < 0.001
ALAN	4.65 ± 0.69	2.84 ± 0.97*	11:58 ± 01:18**	0.71	$F_{(2,33)} = 16.40$	p < 0.001

Rhythm parameters: mesor ± 95% confidence limits (the time series mean), amplitude ± 95% confidence limits (one-half the peak-to-trough difference) and acrophase ± 95% confidence limits (the peak of the fitted curve). R^2, *F*- and *p*-values describe the significance of 24-h rhythms. A rhythm with $p < 0.05$ is considered significant and with a trend to significance if *p*-value is between 0.05 and 0.1, both are indicated with bold font.

Abbrevations: *Bmal1* - brain and muscle ARNT-like 1, *Clock* - circadian locomotor output cycles kaput, *Cry1* – cryptochrome 1, *Per1* – period 1, *Nr1d1* - nuclear receptor subfamily 1 group d member 1 or REV-ERBα

Table S10. The two-way ANOVA of clock genes in rats kept in either the control regime (CTRL) or exposed to dim light at night (ALAN).

	Time	Regime	Interaction
**LIVER**			
Bmal1	$F_{(5,58)}$ = 66.78<br>p < 0.001 ***	$F_{(1,58)}$ = 0.08<br>p = 0.78	$F_{(5,58)}$ = 7.86<br>p < 0.001 ***
Clock	$F_{(5,60)}$ = 40.07<br>p < 0.001 ***	$F_{(1,60)}$ = 0.34<br>p = 0.56	$F_{(5,60)}$ = 0.80<br>p = 0.55
Cry1	$F_{(5,59)}$ = 62.18<br>p < 0.001 ***	$F_{(1,59)}$ = 0.86<br>p = 0.36	$F_{(5,59)}$ = 0.69<br>p = 0.63
Nr1d1	$F_{(5,60)}$ = 151.73<br>p < 0.001 ***	$F_{(1,60)}$ = 7.08<br>p < 0.01 **	$F_{(5,60)}$ = 15.05<br>p < 0.001 ***
Per1	$F_{(5,60)}$ = 117.59<br>p < 0.001 ***	$F_{(1,60)}$ = 1.07<br>p = 0.30	$F_{(5,60)}$ = 1.59<br>p = 0.18
**EPIDIDYMAL FAT**			
Bmal1	$F_{(5,59)}$ = 195.07<br>p <0.001 ***	$F_{(1,59)}$ = 1.80<br>p = 0.19	$F_{(5,59)}$ = 3.08<br>p < 0.05 *
Clock	$F_{(5,59)}$ = 9.76<br>p < 0.001 ***	$F_{(1,59)}$ = 0.54<br>p = 0.47	$F_{(5,59)}$ = 0.18<br>p = 0.97
Cry1	$F_{(5,59)}$ = 53.57<br>p <0.001 ***	$F_{(1,59)}$ = 1.11<br>p = 0.30	$F_{(5,59)}$ = 0.67<br>p = 0.65
Nr1d1	$F_{(5,59)}$ = 63.58<br>p <0.001 ***	$F_{(1,59)}$ = 0.17<br>p = 0.68	$F_{(5,59)}$ = 1.41<br>p = 0.23
Per1	$F_{(5,59)}$ = 16.00<br>p <0.001 ***	$F_{(1,59)}$ = 3.85<br>p = 0.05 #	$F_{(5,59)}$ = 2.98<br>p < 0.05

F- and *P*-values of factors time, regime and their interaction. Significant effects are indicated by * $p < 0.05$, ** $p < 0.01$, *** $p < 0.001$ and # a trend to significance (*p*-value between 0.05 and 0.1). Abbrevations: *Bmal1* - brain and muscle ARNT-like 1, *Clock* - circadian locomotor output cycles kaput, *Cry1* – cryptochrome 1, *Per1* – period 1, *Nr1d1* - nuclear receptor subfamily 1 group d member 1 or REV-ERBα

References

Stenvers, D.J., Van Dorp, R., Foppen, E., Mendoza, J., Opperhuizen, A.-L., Fliers, E., et al. (2016). Dim light at night disturbs the daily sleep-wake cycle in the rat. *Sci Rep* 6**,** 35662. doi: 10.1038/srep35662.
